# Synthesis, Biological Evaluation and Molecular Docking Studies of 6-Aryl-2-Styrylquinazolin-4(3*H*)-Ones

**DOI:** 10.3390/molecules21010028

**Published:** 2015-12-25

**Authors:** Emmanuel Ndubuisi Agbo, Tshepiso Jan Makhafola, Yee Siew Choong, Malose Jack Mphahlele, Ponnadurai Ramasami

**Affiliations:** 1 Department of Chemistry, College of Science, Engineering and Technology, University of South Africa, P. O. Box 392, Pretoria 0003, South Africa; tagboen@unisa.ac.za; 2 Department of Life and Consumer Sciences, College of Agriculture and Environmental Sciences, University of South Africa, Private Bag X06, Florida 1710, South Africa; makhat@unisa.ac.za; 3 Institute for Research in Molecular Medicine (INFORMM), Universiti Sains Malaysia, 11800 Minden, Penang, Malaysia; yeesiew@usm.my; 4 Computational Chemistry Group, Department of Chemistry, Faculty of Science, University of Mauritius, Réduit 80837, Mauritius; ramchemi@intnet.mu

**Keywords:** 6-bromo-2-styrylquinazolin-4(3*H*)-ones, Suzuki-Miyaura cross-coupling, 6-aryl-2-styrylquinazolin-4(3*H*)-ones, *in vitro* cytotoxicity, antimicrobial activity, docking studies

## Abstract

Suzuki-Miyaura cross-coupling of 6-bromo-2-styrylquinazolin-4(3*H*)-ones with arylboronic acids afforded a series of novel 6-aryl-2-styrylquinazolin-4(3*H*)-ones. These compounds were evaluated for potential anticancer properties against the human renal (TK-10), melanoma (UACC-62) and breast cancer (MCF-7) cell lines. Their antimicrobial properties were also evaluated against six Gram-positive and four Gram-negative bacteria, as well as two strains of fungi. Molecular docking studies (*in silico*) were conducted on compounds **5a**, **b**, **d** and **6a**, **b**, **d**–**f** to recognize the hypothetical binding motif of the title compounds within the active site of the dihydrofolate reductase and thymidylate synthase enzymes.

## 1. Introduction

**Figure 1:**
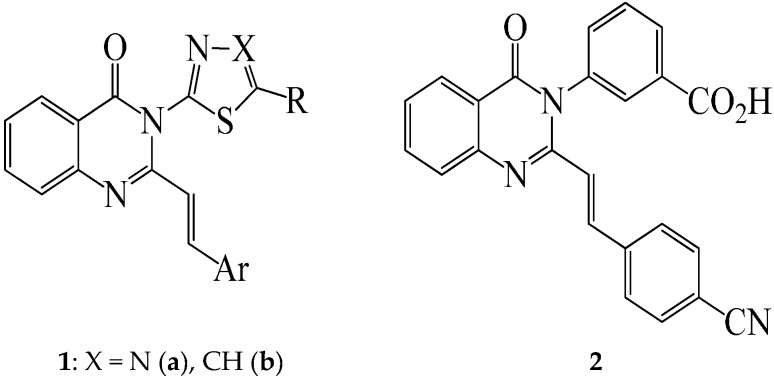
3-substituted 2-styrylquinazolin-4(3*H*)-ones **1** and **2** with antimicrobial activity.

2-Styrylquinazolin-4(3*H*)-one–based compounds continue to attract considerable attention because of their interesting biological activity and pharmacological properties as anticancer [1], anticonvulsant [2], sedative-hypnotic [3,4], antibacterial and antifungal agents [5,6]. A series of 6-substituted 2-styrylquinazolin-4(3*H*)-ones, for example, was evaluated for biological activity as antimitotic agents and the compounds were found to inhibit tubulin polymerization [7]. The presence of a 2-styryl moiety was also found to enhance the antimicrobial activity of quinazolinone derivatives, *viz*., the 3-[5-(4-substituted)phenyl-1,3,4-thiadiazole-2-yl]-2-styrylquinazolin-4(3*H*)-ones **1a** (X = N) [5,6] and the analogous 3-thiazole–substituted 2-styrylquinazolin-4(3*H*)-ones **1b** (X = CH) [8], which exhibit significant antibacterial activity (Figure 1). The analogous (*E*)-3-(3-carboxyphenyl)-2-(4-cyanostyryl)quinazolin-4(3*H*)-one (**2**) is an effective antibiotic *in vivo* against methicillin-resistant *Staphylococcus aureus* (MRSA) and it inhibits cell wall biosynthesis by binding to DD-transpeptidases involved in cross-linking of the cell wall [9]. Extensive structure activity relationship studies on the 3-substituted 2-styrylquinazolin-4(3*H*)-ones revealed that the entire styrylquinazolin-4(3*H*)-one scaffold was required for inhibition of tubulin polymerization [7] and for antimicrobial activity [6,10].

Quinazolinones produce their anticancer activity through inhibition of various enzymes, such as epidermal growth factor receptor tyrosine kinase, dihydrofolate reductase, folate thymidylate synthase, tyrosine kinase, aldose reductase, cyclic GMP phosphodiesterase and DNA repairing enzymes [11]. Raltitrexed (Tomudex™), for example, is a quinazolinone derivative with chemotherapeutic properties known to inhibit dihydrofolate reductase (DHFR) and thymidylate synthase (TS) [12,13], which are the primary targets for the development of new anticancer agents. Some of the compounds that inhibit these enzymes have also been found to exhibit antibiotic and antimalarial properties [14]. DHFR and TS are also often used as models in molecular docking studies to predict the hypothetical protein-ligand binding mode, which plays a significant role in structural based drug design and structure activity relationship. Our interest in the synthesis and biological property studies of polycarbo-substituted quinazolin-4-ones prompted us to investigate the possibility to synthesize novel 2-styrylquinazolinon-4(3*H*)-ones bearing an aryl substituent at the 6-position for further studies of anticancer and antimicrobial activity. Our approach to the requisite 6-aryl-2-styrylquinazolin-4(3*H*)-ones involves palladium catalysed Suzuki-Miyaura cross-coupling of 6-bromo-2-styrylquinazolin-4(3*H*)-ones with arylboronic acids. These polycarbo-substituted quinazolin-4(3*H*)-ones were evaluated for potential *in vitro* cytotoxicity against a panel of cancer cell lines and for *in vitro* antimicrobial activity against both Gram-positive and Gram-negative bacteria as well as fungal strains. Computer docking (*in silico*) studies of the most active compounds were performed against active sites of DHFR and TS to predict the hypothetical protein-ligand binding mode of these compounds as well as to compare binding affinity and conformations with the inhibitor in the crystal structure of DHRF and TS.

## 2. Results and Discussion

### 2.1 Chemistry

**Scheme 1:**
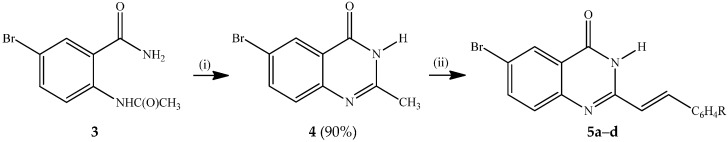
Synthesis of 6-bromo-2-styrylquinazolin-4(3*H*)-ones **5a**–**d**.

Several methods have been developed to date for the synthesis of 2-styrylquinazolin-4(3*H*)-ones and these include the reaction of anthranilic acid with styrylcarboxylic acids followed by amine insertion [1], and Knoevenagel condensation of 2-methyl-3-substituted quinazolin-4(3*H*)-ones with aromatic aldehydes in the presence of a base or acid [7,15,16,17]. Amidation of 2-aminobenzonitrile with 3-arylacryloyl chlorides, followed by oxidative ring closure under basic conditions also afforded 2-styrylquinazolin-4(3*H*)-ones [18]. Kumar *et al.* recently developed a simple, convenient and green synthetic protocol involving a one-pot tandem condensation of substituted isatoic anhydrides, carbo-substituted amines and orthoesters under catalyst- and solvent-free conditions to afford 3-aryl/heteroaryl-2-styrylquinazolin-4(3*H*)-ones [19]. We opted for the method that involves initial acetylation of 2-amino-5-bromobenzamide with acetic anhydride to afford 2-acetamido-5-bromobenzamide (**3**), which upon base-promoted cyclization in ethanol under reflux yielded 6-bromo-2-methylquinazolin-4-one (**4**) (Scheme 1). Knoevenagel condensation of **4** with benzaldehyde derivatives in the presence of acetic acid under reflux afforded the corresponding 2-(arylethenyl)-6-bromoquinazolin-4(3*H*)-ones **5a**–**d**. The structures of compounds **5a**–**d** were assigned based on their NMR, IR and mass spectrometric data. Styrylquinazolines can exist as either *E-* or *Z-*isomers according to the orientation of the ethylene linker, with coupling constant values *J* > 16 Hz or *J* < 12 Hz for the *E* or *Z-*isomers, respectively [19]. The ^1^H-NMR spectra of compounds **3a**–**d** reveal the presence of two doublets at δ *ca.* 6.91 ppm (R = H, F) or 6.99 ppm (R = Cl, OMe) and in the δ 7.91–7.93 ppm region with coupling constant values *J* = 16.0 Hz, which indicates that the double bond exists in an *E*-geometry in agreement with the literature assignment for the analogous compounds [20,21,22,23]. The analogous 3-phenyl/pyridinyl substituted *trans*-2-(aryl/hetearyl)vinylquinazolin-4(3*H*)-ones exhibited significantly reduced emission intensities in solution at room temperature due to competitive quenching through *trans-cis* C=C photo-isomerization [24]. However, these conformational changes were found to be suppressed at 77 K in a frozen glass leading to significantly enhanced emission intensity [24].

*Reagents and conditions*: (i) 5% NaOH (aq), EtOH, reflux, 1 h; (ii) RC_6_H_4_CHO, AcOH, reflux, 6 h.

**Table:** 

Compound	R	%Yield of 5
**5a**	4-H	73
**5b**	4-F	65
**5c**	4-Cl	68
**5d**	3-OMe	71

**Scheme 2:**
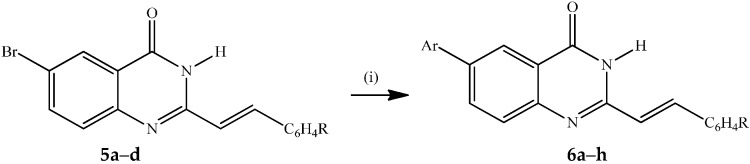
Suzuki-Miyaura cross-coupling of **5a**–**d** to afford **6a**–**h**.

The 6-bromoquinazolin-4(3*H*)-ones **5a**–**d** were subjected to the Suzuki-Miyaura cross-coupling with arylboronic acids in the presence of PdCl_2_(PPh_3_)_2_ as the source of active Pd(0) species and potassium carbonate as a base in 3:1 dioxane–water (*v*/*v*) under reflux for 3 h to afford the corresponding novel 6-aryl–substituted derivatives **6a**–**h** (Scheme 2).

Reagents *and* conditions: (i) ArB(OH)_2_ (1.2 equiv.), PdCl_2_(PPh_3_)_2_, K_2_CO_3_ (3 equiv.), dioxane–H_2_O (3:1, *v*/*v*), reflux, 3 h.

**Table:** 

Compound	R	Ar	%Yield of 6
**6a**	4-H	-C_6_H_5_	61
**6b**	4-F	-C_6_H_5_	68
**6c**	4-Cl	-C_6_H_5_	71
**6d**	3-OMe	-C_6_H_5_	75
**6e**	4-H	4-FC_6_H_4_-	66
**6f**	4-F	4-FC_6_H_4_-	63
**6g**	4-Cl	4-FC_6_H_4_-	65
**6h**	3-OMe	4-FC_6_H_4_-	72

The 6-carbo–substituted 2-styrylquinazolin-4(3*H*)-ones **6a**–**h** do not dissolve in most commonly used deuterated solvents and are only sparingly soluble in DMSO-*d*_6_ to enable acquisition of the ^1^H-NMR spectra within a few minutes. Some of the compounds, e.g., **6c** and **6g** were found to precipitate from DMSO-*d*_6_ at room temperature and this behaviour compromised the quality of their ^13^C-NMR spectra. These compounds are easily distinguished from the corresponding precursors by the increased number of proton signals in the aromatic region of their ^1^H-NMR spectra. Moreover, the accurate calculated *m*/*z* values represent close fits consistent with the assigned structures. As a guide to quinazolinone derivatives with potential biological properties, we decided to evaluate the 6-bromo-2-styrylquinazolinones **5a**–**d** and the 6-aryl-2-styrylquinazolin-4(3*H*)-ones **6a**–**h** for *in vitro* cytotoxicity against a panel of cancer cell lines and for antimicrobial activity against the Gram-positive and Gram-negative bacteria, as well as fungi.

### 2.2. Biological Properties

#### 2.2.1. *In vitro* Cytotoxicity Studies of **5a**–**d** and **6a**–**h**

The 6-bromoquinazolinones **5a**–**d** and their derivatives **6a**–**h** were evaluated for their *in vitro* cytotoxicity against the human renal (TK-10), melanoma (UACC-62) and breast cancer (MCF-7) cell lines using the sulforhodamine B stain (SRB) assay [25] to correlate between both structural variations and cytotoxic activity of the synthesized compounds. The IC_50_ values of the tested compounds **5a**–**d** and **6a**–**h** against human renal (TK-10), melanoma (UACC-62) and breast cancer (MCF-7) cell lines at concentrations 0.01, 0.1, 1.0, 10.0 and 100 μM using parthenolide as reference drug are represented in Table 1 (see Supplementary Materials for the corresponding cell viability percentages and graphs for each compound). Increased potency against the three cell lines was observed for the 6-bromo-2-styrylquinazolin-4(3*H*)-ones **5a** (R = 4-H), **5b** (R = 4-F) and **5d** (R = 3-OCH_3_). Moderate activity was observed for the 2-(4-chlorostyryl) derivative **3c** against human renal (IC_50_ = 22.48 μM) and melanoma (IC_50_ = 14.96 μM) cell lines with no activity against the breast cancer (MCF-7) cell line. Replacement of bromine with phenyl ring diminished activity against all the cell lines for majority of the 6-phenyl-2-styrylquinazolin-4-ones. Compound **6a** showed selectivity and moderate activity against the melanoma (UACC-62) and breast cancer (MCF-7) cell lines with IC_50_ values of 22.99 μM and 37.18 μM, respectively. Moderate activity against breast cancer (MCF-7) cell line was also observed for the 2-(3-methoxystyryl) derivative **6d** with IC_50_ value of 25.23 μM. Replacement of 6-Br with 4-fluorophenyl group, on the other hand, resulted in diminished activity against all the three cell lines except for the 2-(4-fluorostyryl) derivatives **6f**, which showed moderate cytotoxicity against melanoma (UACC-62) and breast cancer (MCF-7) cell lines with IC_50_ value of 19.70 μM and 29.26 μM, respectively. In general, the 6-bromo-2-styrylquinazolinon-4-ones exhibit enhanced inhibitory activity than the corresponding 6-aryl–substituted derivatives. Literature precedents for analogous compounds demonstrated that inhibitory activity increases with decreasing size of the hydrophobic substituents at position 6 [1].

Compounds that have both anticancer and antimicrobial activity have promising therapeutic potential due to their selective cytotoxicity coupled with the potential to reduce the occurrence of bacterial and fungal infections in immune-compromised cancer patients [26]. To evaluate the potential of compounds **5a**–**d** and **6a**–**h** to serve as dual anticancer and antimicrobial agents, we also evaluated them for *in vitro* antimicrobial activity against ATCC bacterial and fungal strains representing pathogenic species of different classes commonly associated with nosocomial infections [26].

**Table 1:** IC_50_ values of the test compounds against the three cancer cell lines. 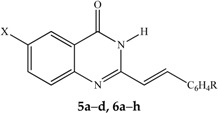

Compound	X	R	IC_50_ (µM) TK-10	IC_50_ (µM) UACC-62	IC_50_ (µM) MCF-7
**5a**	Br	4-H	7.72 ± 0.08	1.98 ± 1.89	2.12 ± 0.20
**5b**	Br	4-F	2.42 ± 0.07	1.03 ± 0.19	3.47 ± 0.013
**5c**	Br	4-Cl	22.48 ± 0.48	14.95 ± 2.67	100 ± 0.00
**5d**	Br	3-OMe	1.12 ± 0.05	0.62 ± 0.04	1.80 ± 0.30
**6a**	-C_6_H_5_	4-H	100 ± 0.00	22.99 ± 1.03	37.18 ± 3.36
**6b**	-C_6_H_5_	4-F	100 ± 0.00	42.44 ± 8.59	48.83 ± 4.93
**6c**	-C_6_H_5_	4-Cl	100 ± 0.00	100 ± 0.00	100 ± 0.00
**6d**	-C_6_H_5_	3-OMe	100 ± 0.00	44.68 ± 13.72	25.23 ± 0.87
**6e**	4-FC_6_H_4_-	4-H	100 ± 0.00	96.31 ± 5.22	77.41 ± 26.01
**6f**	4-FC_6_H_4_-	4-F	100 ± 0.00	19.70 ± 3.19	29.26 ± 4.99
**6g**	4-FC_6_H_4_-	4-Cl	100 ± 0.00	100 ± 0.00	100 ± 0.00
**6h**	4-FC_6_H_4_-	3-OMe	100 ± 0.00	96.31 ± 5.22	77.41 ± 26.01
**Parthenolide**			1.56 ± 0.25	3.26 ± 0.40	3.68 ± 0.01

#### 2.2.2. *In vitro* Antimicrobial Activity of **3a**–**d** and **4a**–**h**

The *in vitro* antimicrobial activities of compounds **3a**–**d** and **4a**–**h** were tested by determining the minimal inhibitory concentrations (MIC) using the microplate serial dilution assay [27] with slight modifications for fungi [28]. A total of ten bacterial pathogens (nine ATCC strains and one CSIR isolate) and two ATCC fungal pathogens were selected as test organisms in this study. The microorganisms used in this study are ATCC strains and represent pathogenic species of different classes commonly associated with nosocomial infections [26]. For both the antibacterial and antifungal assays, the MIC values were determined visually after 24 h of incubation with the compounds to determine if these compounds possess either bacteriostatic or bactericidal activity and either fungistatic or fungicidal activity. The MIC was recorded as the lowest concentration of the compounds at which the bacterial or fungal growth was inhibited. The test compounds have varying antimicrobial activity in both the antibacterial and antifungal assays, and the results are presented in Table 2 and Table 3.

**Table 2:** Minimum inhibitory concentration (MIC, µg/mL) of **5a**–**d** and **6a**–**h** against Gram-positive and Gram-negative bacterial strains

Compound	*B. c*	*E. h*	*E. f*	*E. g*	*E. c*	*S. a*	*A. c*	*E. c*	*P. a*	*S. t*
**5a**	15.62	15.62	31.25	7.81	15.62	31.25	62.50	31.25	31.25	31.25
**5b**	7.81	15.62	31.25	7.81	62.50	31.25	62.50	62.50	15.62	125
**5c**	62.5	62.50	31.25	7.81	62.50	˃250	125	125	31.25	125
**5d**	31.25	31.25	31.25	31.25	˃250	62.50	62.50	62.50	62.50	31.25
**6a**	15.62	15.62	15.62	15.62	31.25	31.25	62.50	62.50	31.25	62.5
**6b**	15.62	15.62	15.62	15.62	31.25	31.25	31.25	31.25	31.25	15.62
**6c**	125	31.25	62.50	125	125	62.50	31.25	˃250	62.50	7.81
**6d**	31.25	31.25	125	125	125	˃250	˃250	˃250	˃250	31.25
**6e**	125	31.25	31.25	62.50	62.50	31.25	62.50	62.50	62.50	15.62
**6f**	62.50	62.50	125	31.25	˃250	˃250	˃250	62.50	125	15.62
**6g**	62.50	62.50	62.50	31.25	125	125	˃250	62.50	31.25	125
**6h**	˃250	62.50	˃250	˃250	31.25	62.50	62.50	31.25	125	125
**Gentamicin**	3.12	1.56	6.25	1.56	1.56	0.78	1.56	0.39	6.25	0.78

*B. c = Bacillus cereus*; *E. h = Enterococcus hirae*; *E. f = Enterococcus faecalis*; *E. g = Enterococcus gallinarium*; *E. c = Enterococcus casseliflavus*; *S. a = Staphylococcus aureus*; *A. c = Acinetobacter calcaoceuticals anitratus*; *E. c = Escherichia coli*; *P. a = Pseudomonas aeruginosa*; *S. t = Salmonella typhi*.

**Table 3:** Minimum inhibitory concentration (MIC, µg/mL) of **5a**–**d** and **6a**–**h** against fungal strains.

Compound	*Candida albicans*	*Cryptococcus neoformans*
**5a**	15.62	15.62
**5b**	31.25	31.25
**5c**	31.25	31.25
**5d**	31.25	31.25
**6a**	62.50	31.25
**6b**	31.25	31.25
**6c**	62.50	31.25
**6d**	31.25	62.50
**6e**	62.50	31.25
**6f**	31.25	62.50
**6g**	62.50	62.50
**6h**	31.25	15.62
**Amphotericin-B**	3.90	1.95

Moderate growth inhibitory activity against all the Gram-positive bacteria was observed for the 6-bromoquinazolinones **5a**, **5b** and **5c**. Substitution of bromine with phenyl or 4-fluorophenyl group led to loss of activity for compounds **6a**–**h**. Less or no activity was observed against the Gram-negative bacterial strains for these compounds in analogy with literature observation for the 3-[5-(4-substituted)phenyl-1,3,4-thiadiazole-2-yl]-2-styrylquinazolin-4(3*H*)-ones activity [8]. Gram-negative bacterial strains are surrounded by an additional outer membrane layer, which presumably presents a barrier to penetration or entry of noxious compounds [29]. Both series of compounds were found to exhibit moderate inhibitory activity against the fungal strains. 

In the last part of this investigation, we performed computer docking (*in silico)* studies on the most active compounds **5**(**a**, **b**, **d**) and **6**(**a**, **b**, **d**–**f**) against active sites of DHFR and TS using raltitrexed as a standard to predict the hypothetical protein-ligand binding mode of these compounds.

#### 2.2.3. Molecular Docking Studies

**Figure 2:**
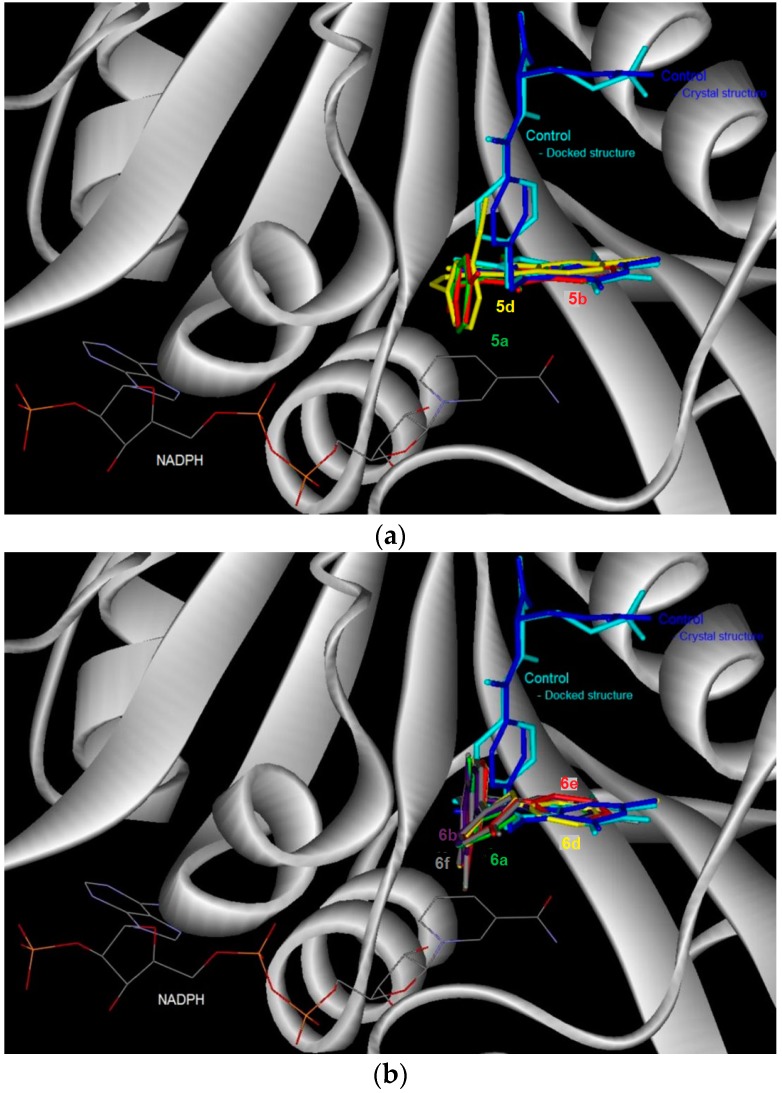
The binding conformation in human dihydrofolate reductase (DHFR) for the 6-bromoquinazolinones (**a**) and their 6-aryl–substituted derivatives (**b**).

**Figure 3:**
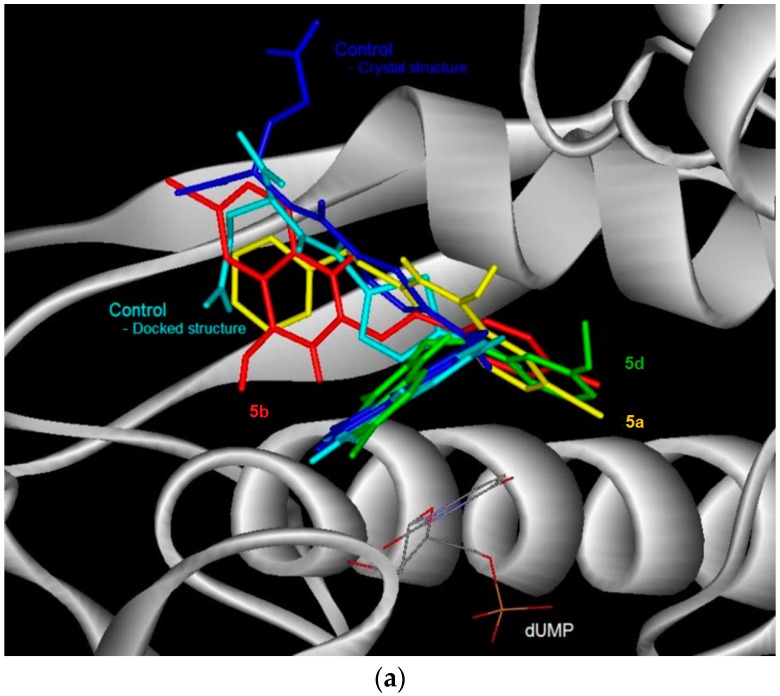

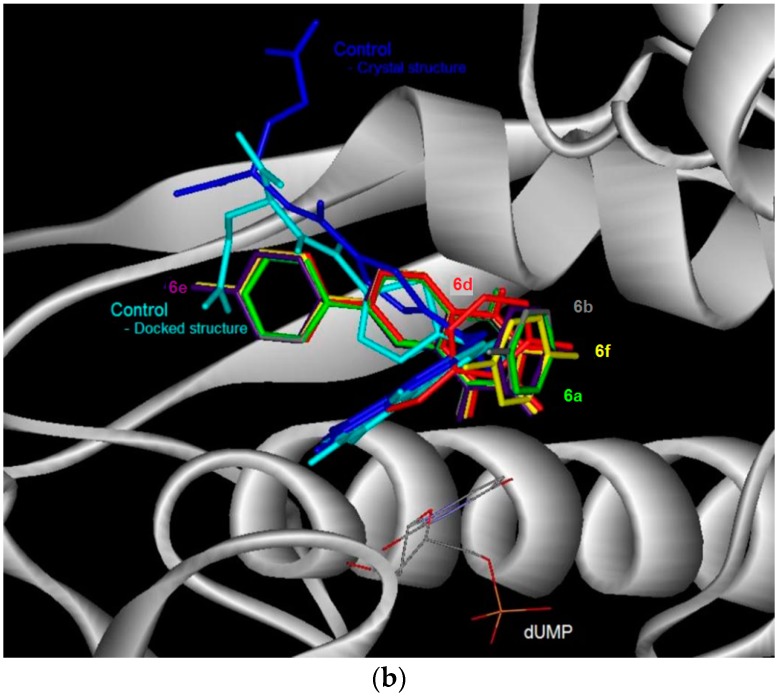
The binding conformation in human thymidylate synthase (TS) for the 6-bromoquinazolinones (**a**) and their 6-aryl substituted derivatives (**b**).

Control docking of the co-factor for DHFR and TS showed that docking simulations (Figure 2 and Figure 3, Table 4) were able to reproduce the experimental data where the docked conformations have the root mean square deviation (RMSD) from the crystal structure of only 0.5 and 1.7 Å, respectively. In general, the 6-bromoquinazolinones **5a**, **5b** and **5d** have the more favorable binding affinity with DHRF compared with that of TS. A total of five hydrogen bond interactions between the inhibitor (*N*-{4-[(2-amino-6-ethyl-4-oxo-3,4-dihydrothieno[2,3-*d*]pyrimidin-5-yl)thio]benzoyl}-l-glutamic acid) with DHFR were detected in the crystal structure of DHFR (Table 4). This has thus explained the most favorable binding affinity of the inhibitor with DHFR compared with that of the test compounds. It is also interesting to note hydrogen bonding was not formed between carbo-substituted derivatives **6a**, **6b** and **6d**–**f** with DHRF. These carbo-substituted derivatives could interact with DHFR through other non-bonded in4teractions such as van der Waals and electrostatic interactions, hydrophobic contacts and aromatic-aromatic stacking interactions. The binding energy for the carbo-substituted derivatives **6a**, **6b** and **6d**–**f** were similar with that of the 6-bromoquinazolinones. Except **5a**, 1-3 hydrogen bonding was formed between **5b**, **5d**, **6a**, **6b**, **6d**–**f** with TS. The most favorable binding free energy of **4a** with TS compared with other compounds might also be due to contribution by other non-bonded interactions besides hydrogen bonding. Compound **5b** exhibits significant binding affinity towards DHRF and TS, respectively. Taking into consideration of both binding free energy and hydrogen bonding, the 6-bromoquinazolinones (**5a**, **5b**, **5d**) would exhibit significant inhibitory activity against DHFR, while their 6-aryl–substituted derivatives (**6a**, **6b**, **6d**–**f**) display potential to inhibit TS. The 6-bromoquinazolinones and their substituted derivatives appear to bind at the same binding cavity with the inhibitor (*N*-{4-[(2-amino-6-ethyl-4-oxo-3,4-dihydrothieno[2,3-*d*]pyrimidin-5-yl)thio]benzoyl}-l-glutamic acid) of DHRF (Figure 2) and TS (Figure 3) as observed in the crystal structure. In addition, from the binding affinity, the test compounds could inhibit better for DHRF than TS. It is noticed that the benzene ring of all the compounds was pointing towards the exit of the binding cavity in DHFR. As compared with the classical antifolate in the crystal structure of DHFR, its carboxylic tail was orientated toward the hydrophilic helix formed by Arg28-Thr40. This might contribute to a better interaction of this antifolate with DHFR, thus explain the more favourable binding affinity (<−13 kcal/mol) compared with the test compounds (*ca*. −10 kcal/mol). Favourable aromatic-aromatic stackings (π-π and π-σ interactions) were observed between the quinazolinone ring of the test compounds with the pyridine ring of the co-factor for DHRF. It is also noticed that interactions of the quinazolinone ring docked at similar position with the thieno[2,3-*d*]pyrimidine of the *N*-{4-[(2-amino-6-ethyl-4-oxo-3,4-dihydrothieno[2,3-*d*]pyrimidin-5-yl)thio]benzoyl}-l-glutamic acid in the crystal structure of DHFR [12].

The docked conformation of the test compounds was comparable with the inhibitor (Tomudex) of the crystal of thymidylate synthase (TS) [13]. The benzene ring of all the test compounds was pointing towards the C-terminus of TS. While the carboxylic tail of Tomudex was pointing towards the exit of the binding cavity in TS which might not increase the interaction with TS. Therefore, it could be explained that the binding affinity of Tomudex is comparable with the test compounds. Favorable aromatic-aromatic stacking (π-π and π-σ interactions) were also observed between the quinazolinone ring of 6-bromoquinazolinones compounds with the pyrimidine ring of the cofactor (dUMP) for TS. However, this aromatic-aromatic stacking was not observed for the 6-aryl-quinazolinone derivatives.

**Table 4:** Molecular docking results of test compounds against human dihydrofolate reductase (DHFR) and thymidylate synthase (TS).

	Dihydrofolate Reductase (DHFR)		Thymidylate Synthase (TS)	
Compound	Binding Free Energy (kcal/mol)	No. Hydrogen Bond & Distance (Å)	Binding Free Energy (kcal/mol)	Hydrogen Bond
**5a**	−10.36	2	−7.71	0
		(i) H(**5a**)-O(Val115): 2.16		
		(ii) H_O_(**5a**)-O(Ile7): 1.85		
**5b**	−10.33	3	−8.08	3
				(i) H_Z1_(Lys77)-O(**5b**): 2.16
				(ii) H_O_(**5b**)-O(Leu221): 2.19
		(i) H(**5b**)-O(Val115): 2.14		(iii) H(**5b**)-O(Leu221): 2.17
		(ii) H_H_(Tyr121)-O(**5b**): 2.46		
		(iii) H_O_(**5b**)-O(Ile7): 1.79		
**5d**	−10.86	2	−7.66	1
		(i) H(**5d**)-O(Val115): 1.90		(i) H(Gly222)-O(**5d**): 1.79
		(ii) H_H_(Tyr121)-O(**5d**): 2.47		
**6a**	−10.32	0	−9.14	2
				(i) H_D22_(Asn226)-O(**6a**): 2.48
				(ii) H(**6a**)-O_4_(UMP): 2.11
**6b**	−10.24	0	−9.07	1
				(i) H_D22_(Asn226)-O(**6b**): 2.48
**6d**	−10.78	0	−8.89	2
				(i) H_D22_(Asn226)-O(**6d**): 2.35
				(ii) H(**6b**)-O_4_(UMP): 2.11
**6e**	−10.10	0	−9.08	2
				(i) H_D22_(Asn226)-O(**6e**): 2.31
				(ii) H(**6e**)-O_4_(UMP): 1.97
**6f**	−10.84	0	−9.00	2
				(i) H_D22_(Asn226)-O(**6f**): 2.35
				(ii) H(**6f**)-O_4_(UMP): 2.09
**Control (Ctrl)**	−13.09	5	−9.07	3
				(i) H(Phe80)-O_E1_(**Ctrl**): 2.25
		(i) H_H22_(Arg70)-O_AG_(**Ctrl**): 1.69		(ii) HZ2(Lys77)- O_E1_(**Ctrl**): 2.03
		(ii) H_H22(_Arg70)-O_AD_(**Ctrl**): 2.66		(iii) H_3_(**Ctrl**)-O_D2_(Asp218): 1.80
		(iii) H_H12_(Arg70)-O_AD_(**Ctrl**): 1.83		
		(iv) H_D21_(Asn64)-O_AE_(**Ctrl**): 2.27		
		(v) (Try121) H_H_-S_4_(**Ctrl**): 3.23		
		(v) H_1_(**Ctrl**)-O_E2_(Glu): 2.03		

## 3. Materials and Methods

### 3.1. General Information

Melting points were recorded on a Thermocouple digital melting point apparatus and are uncorrected. IR spectra were recorded as powders using a Bruker VERTEX 70 FT-IR Spectrometer (Bruker Optics, Billerica, MA, USA) with a diamond attenuated total reflectance (ATR) accessory by using the thin-film method. For column chromatography, Kieselgel 60 (0.063–0.200 mm) (Merck KGaA, Frankfurt, Germany) was used as stationary phase. NMR spectra were obtained as CDCl_3_ or DMSO-*d*_6_ solutions using Mercury 300 MHz (Varian Inc., Palo Alto, CA, USA) or Agilent 500 MHz NMR (Agilent Technologies, Yarnton, UK) spectrometers and the chemical shifts are quoted relative to the TMS peak. Low- and high-resolution mass spectra were recorded at an ionization potential of 70 eV using Synapt G2 Quadrupole 6 Time-of-flight mass spectrometer (Waters Corp., Milford, MA, USA) at the University of Stellenbosch Mass Spectrometry Unit. The synthesis and analytical data of compound **3** has been described before [30]. The ^1^H- and ^13^C-NMR spectra of compounds **4**–**6 **are listed in the Supplementary Materials.

### 3.2. Synthesis of 6-Bromo-2-Methylquinazolin-4(3H)-One *(**4**)*

A stirred mixture of 2-acetamido-5-bromobenzamide **3** (0.30 g, 1.16 mmol) and 5% NaOH (20 mL) in ethanol (10 mL) was refluxed for 1 h and then allowed to cool to 0 °C. The mixture was acidified dropwise with concentrated hydrochloric acid. The resulting precipitate was filtered on a sintered funnel, washed sequentially with cold water and an ice-cold ethanol and then dried in an oven to afford compound (**4**) as a white solid (0.25 g, 89%), mp. 299–302 °C; ν_max_ (ATR) 495, 777, 898, 1302, 1469, 1641 cm^−1^; δ_H_ (500 MHz, DMSO-*d*_6_) 2.33 (3H. s, -CH_3_), 7.51 (1H, d, *J* = 9.0 Hz, 8-H), 7.90 (1H, dd, *J* = 2.0 and 9.0 Hz, 7-H), 8.12 (1H, d, *J* = 2.0 Hz, 5-H), 12.38 (1H, s, NH); δ_C_ (125 MHz, DMSO-*d*_6_) 21.9, 118.6, 122.7, 128.2, 129.5, 137.5, 148.4, 155.5, 161; *m*/*z* 239 (100, MH^+^); HRMS (ES): MH^+^ found 238.9820. C_9_H_8_N_2_O^79^Br^+^ requires 238.9812.

### 3.3. Typical Procedure for the Synthesis of the 6-Bromo-2-(styryl)Quinazolin-4(3H)-Ones ***5a**–**d***

A stirred mixture of **4** (1 equiv.) and benzaldehyde derivative (1.2 equiv.) in acetic acid (20 mL/mmol of **4**) was refluxed for 6 h. The mixture was allowed to cool and then quenched with an ice-cold water. The resultant precipitate was filtered and washed with methanol to afford compound **5**. The following compounds were prepared in this fashion:

*(E)-6-Bromo-2-(phenylstyryl) quinazolin-4(3H)-one *(**5a**). Solid (0.30 g, 74%), mp. 328–330 °C (Lit. [7] 334–338 °C); ν_max_ (ATR) 534, 827, 967, 1308, 1463, 1667, 3174 cm^−1^; δ_H_ (300 MHz, DMSO-*d*_6_) 6.98 (1H, d, *J*_trans_ = 16.0 Hz, H_a_), 7.40–7.48 (3H, m, ArH), 7.61 (1H, d, *J* = 8.7 Hz, 8-H), 7.64 (2H, dd, *J* = 1.8 and 8.4 Hz, ArH), 7.92 (1H, dd, *J =* 2.5 and 8.7 Hz, 7-H), 7.95 (1H, d, *J*_trans_ = 16.0 Hz, H_b_), 8.16 (1H, d, *J = *2.5 Hz, 5-H), 12.49 (1H, s, NH); δ_C_ (75 MHz, DMSO-*d*_6_) 119.0, 121.2, 123.2, 128.1, 128.4, 129.5, 129.9, 130.4, 135.3, 137.8, 139.3, 148.5, 152.4, 161.1; *m*/*z* 329 (100, MH^+^); HRMS (ES): MH^+^, found 329.0130. C_16_H_12_N_2_O^79^Br^+^ requires 329.0133.

*(E)-6-Bromo-2-(4-fluorostyryl)quinazolin-4(3H)-one* (**5b**). Solid (0.34 g, 78.3%), mp. 332–334 °C; ν_max_ (ATR) 506, 643, 817, 967, 1233, 1463, 1673, 3174 cm^−1^; δ_H_ (500 MHz, DMSO-*d*_6_) 6.91 (1H, d, *J*_trans_ = 16.0 Hz, H_a_), 7.28 (2H, t, *J* = 8.5 Hz, 3′,5′-H), 7.58 (1H, d, *J* = 8.0 Hz, 8-H), 7.71 (2H, t, *J* = 8.5 Hz, 2′,6′-H), 7.90 (1H, dd, *J* = 2.5 and 8.5 Hz, 7-H), 7.93 (1H, d, *J*_trans_ = 16.0 Hz, H_b_), 8.14 (1H, d, *J* = 2.5 Hz, 5-H), 12.48 (1H, s, NH); δ_C_ (125 MHz, DMSO-*d*_6_) 116.6 (d, ^2^*J*_CF_ = 21.8 Hz), 119.0, 121.1, 123.1, 128.4, 129.9, 130.4 (d, ^3^*J*_CF_ = 8.5 Hz), 131.9 (d, ^4^*J*_CF_ = 3.1 Hz), 137.8, 138.1, 148.4, 152.4, 161.0, 163.4 (d, ^1^*J*_CF_ = 246.5 Hz); *m*/*z* 345 (100, MH^+^); HRMS (ES): MH^+^, found 345.0036. C_16_H_11_N_2_O^79^BrF^+^ requires 345.0039.

*(E)-6-Bromo-2-(4-chlorostyryl)quinazolin-4(3H)-one* (**5c**). Solid (0.34 g, 76%) mp. > 345 °C (Lit. [31] > 280 °C); ν_max_ (ATR) 492, 624, 808, 831, 967, 1088, 1306, 1463, 1577, 1645, 1671, 3173 cm^−1^; δ_H_ (500 MHz, DMSO-*d*_6_) 6.98 (1H, d, *J*_trans_ = 16.0 Hz, H_a_), 7.51 (2H, d, *J = *8.5 Hz, 3′,5′-H), 7.60 (1H, d, *J* = 8.5 Hz, 8-H), 7.66, (2H, d, *J* = 8.5 Hz, 2′,6′-H), 7.91 (1H, dd, *J* = 2.5 and 8.5 Hz, 7-H), 7.92 (1H, d, *J* = 16.0 Hz, H_b_), 8.16 (1H, d, *J* = 2.5 Hz, 5-H), 12.50 (1H, s, NH); δ_C_ (125 MHz, DMSO-*d*_6_) 119, 122.1,123.1,128.4, 129.6, 129.8, 129.9, 134.3, 134.7, 137.9, 137.8, 148.3, 152.4, 161.1; *m*/*z* 361 (100, MH^+^); HRMS (ES): MH^+^, found 360.9746. C_16_H_11_N_2_O^35^Cl^79^Br^+^ requires 360.9743.

*(E)-6-Bromo-2-(3-methoxystyryl)quinazolin-4(3H)-one *(**5d**). Solid (0.32 g, 71%), mp. 287–288 °C (Lit. [31] 268–270 °C); ν_max_ (ATR) 535, 775, 890, 976, 1464, 1586, 1676, 3173 cm^−1^; δ_H_ (500 MHz, DMSO-*d*_6_) 3.80 (3H, s, -OCH_3_), 6.98 (1H, dd, *J* = 2.5 and 7.5 Hz, 4′-H), 6.99 (1H, d, *J*_trans_ = 16.5 Hz, H_a_), 7.20 (1H, d, *J* = 2.5 Hz, 2′-H), 7.22 (1H, d, *J* = 7.5 Hz, 6′-H), 7.36 (1H, t, *J* = 8.5 Hz, 5′-H), 7.59 (1H, d, *J* = 8.5 Hz, 8-H), 7.91 (1H, d, *J*_trans_ = 16.0 Hz, H_b_), 7.93 (1H, dd, *J* = 2.5 and 8.5 Hz, 7-H), 8.16 (1H, d, *J* = 2.5 Hz, 5-H), 12.47 (1H, s, NH); δ_C_ (125 MHz, DMSO-*d*_6_) 55.6, 113.1, 116.2, 119.0, 120.5, 121.6, 128.4, 129.9, 130.6, 136.7, 137.8, 139.2, 148.4, 152.4, 160.1 (2xC), 161.0; *m*/*z* 357 (100, MH^+^); HRMS (ES): MH^+^, found 357.0235. C_17_H_14_N_2_O_2_^79^Br^+^ requires 357.0239.

### 3.4. Typical Procedure for the Suzuki-Miyaura Cross-Coupling of ***5a**–**d*** with Arylboronic Acids

*(E)-6-Phenyl-2-styrylquinazolin-4(3H)-one *(**6a**). A stirred mixture of **5a** (0.20 g, 0.61 mmol), PdCl_2_(PPh_3_)_2_ (0.02 g, 0.03 mmol), PCy_3_ (0.017 g, 0.06 mmol) and K_2_CO_3_ (0.10 g, 0.73 mmol) in 3:1 dioxane–water (*v*/*v*; 10 mL) was purged with argon gas for 30 minutes. Phenylboronic acid (0.11 g, 0.91 mmol) was added to the mixture using a syringe. The reaction mixture was heated at 100 °C for 2 h and then quenched with an ice-cold water. The product was extracted into chloroform and the combined organic layers were washed with water, dried over Na_2_SO_4_ and filtered dry on a sintered funnel to afford **6a** as a yellow solid (0.12 g, 61%), mp. 329 °C; ν_max_ (ATR) 491, 695, 753, 794, 966, 1259, 1473, 1579, 1655 cm^−1^; δ_H_ (500 MHz, DMSO-*d*_6_) 7.01 (1H, d, *J*_trans_ = 16.0 Hz, H_a_), 7.40–7.52 (6H, m, ArH), 7.66 (2H, d, *J* = 7.5 Hz, ArH), 7.76 (3H, t, *J* = 7.5 Hz, ArH), 7.96 (1H, d, *J*_trans_ = 16.0 Hz, H_b_), 8.13 (1H, dd, *J* = 2.5 and 7.5 Hz, 7-H), 8.31 (1H, d, *J = *2.5 Hz, 5-H), 12.41 (1H, s, NH); δ_C_ (125 MHz, DMSO-*d*_6_) 121.5, 121.9, 123.6, 127.2 (2xC), 128.1, 128.4, 128.6, 128.7, 130.3, 133.5, 135.4, 138.3, 138.9, 139.3, 148.8, 151.9, 162.3; *m*/*z* 325 (100, MH^+^); HRMS (E) (ES): MH^+^ found 325.1341. C_22_H_17_N_2_O^+^ requires 325.1339.

*(E)-2-(4-Fluorostyryl)-6-phenylquinazolin-4(3H)-one *(**6b**). Solid (0.13 g, 66%), mp. 345 °C; ν_max_ (ATR) 499, 698, 761, 824, 968, 1157, 1552, 1660 cm^−1^; δ_H_ (300 MHz, CDCl_3_) 7.00 (1H, d, *J*_trans_ = 16.0 Hz, H_a_), 7.31 (2H, t, *J* = 9.0 Hz, 2′,6′-H), 7.43 (1H, d, *J* = 7.5 Hz, 8-H), 7.52 (2H, t, *J* = 9.0 Hz, 3′,5′-H), 7.71–7.80 (5H, m, Ph), 7.96 (1H, d, *J*_trans_ = 16.0 Hz, H_b_), 8.13 (1H, dd, *J = *2.0 and 9.0 Hz, 7-H), 8.33 (1H, d, *J* = 2.0 Hz, 5-H), 12.39 (1H, s, NH); δ_C_ (75 MHz, DMSO-*d*_6_) 116.6 (d, ^2^*J*_CF_ = 21.9 Hz), 118,6, 119.0, 121.5, 123.1, 128.2, 128.4, 129.5, 130.4 (d, ^3^*J*_CF_ = 8.5 Hz), 132.0 (d, ^4^*J_CF_* = 3.2 Hz), 137.6, 137.8, 138.1, 148.4, 152.4, 161.1, 163.4 (d, ^1^*J*_CF_ = 246.6 Hz); *m*/*z* 343 (100, MH^+^); HRMS (ES): MH^+^ found 343.1239. C_22_H_16_N_2_OF^+^ requires 343.1247.

*(E)-2-(4-Chlorostyryl)-6-phenylquinazolin-4(3H)-one *(**6c**). Solid (0.22 g, 71%), mp. 345 °C; ν_max_ (ATR) 490, 697, 764, 793, 969, 1652 cm^−1^; δ_H_ (500 MHz, DMSO-*d*_6_) 7.01 (1H, d, *J*_trans_ = 16.0 Hz, H_a_), 7.40 (1H, d, *J* = 7.5 Hz, 8-H), 7.48–7.520 (4H, m, ArH), 7.67 (2H, d, *J* = 8.0 Hz, 3′,5′-H), 7.74 (1H, d, *J* 7.5 Hz, 8-H), 7.75 (2H, d, *J* = 8.5 Hz, 2′,6′-H), 7.93 (1H, d, *J*_trans_ = 16.5 Hz, H_b_), 8.12 (1H, dd, *J* = 2.0 and 7.5 Hz, 7-H), 8.32 (1H, d, *J* = 2.0 Hz, 5-H), 12.41 (1H, s, NH); *m*/*z* 359 (100, MH^+^); HRMS (E)(ES): MH^+^ found 359.094. C_22_H_16_N_2_O^35^Cl^+^ requires 359.095.

*(E)-2-(3-Methoxystyryl)-6-phenylquinazolin-4(3H)-one* (**6d**). Solid (0.23 g, 75%), mp. 268 °C; ν_max_ (ATR) 529, 827, 871, 1156, 1226, 1310, 1474, 1582, 1661, 3176 cm^−1^; δ_H_ (500 MHz, DMSO-*d*_6_) 3.80 (3H, s, -OCH_3_), 6.98 (1H, dd, *J* = 1.5 and 7.5 Hz, 6'-H), 7.02 (1H, d, *J*_trans_ = 16.0 Hz, H_a_), 7.22 (1H, d, *J* = 1.5 Hz, 2'-H), 7.23 (1H, d, *J* = 7.5 Hz, 4′-H), 7.36 (1H, t, *J* = 9.0 Hz, 5′-H), 7.41 (1H, d, *J* = 7.5 Hz, 8-H), 7.50 (2H, t, *J* = 9.0 Hz, ArH), 7.73–7.77 (3H, m, ArH), 7.93 (1H, d, *J*_trans_ = 16.0 Hz, H_b_), 8.11 (1H, dd, *J* = 2.0 and 7.5 Hz, 7-H), 8.31 (1H, d, *J* = 2.0 Hz, 5-H), 12.38 (1H, s, NH); δ_C_ (125 MHz, DMSO-*d*_6_) 55.6, 113.1, 116.1, 120.5, 121.8, 121.9, 123.6, 127.2 (2xC), 128.3, 129.6, 130.5, 133.4, 136.9, 138.3, 138.7, 139.3, 148.7, 151.8, 160.2, 162.1; *m*/*z* 355 (100, MH^+^); HRMS (ES): MH^+^ found 355.1435. C_23_H_19_N_2_O_2_^+^ requires 355.1447.

*(E)-6-(4-Fluorophenyl)-2-styrylquinazolin-4(3H)-one* (**6e**). Solid (0.20 g, 66%), mp. 343 °C; ν_max_ (ATR) 490, 528, 696, 755, 968, 1156, 1226, 1478, 1661 cm^−1^; δ_H_ (500 MHz, DMSO-*d*_6_) 7.01 (1H, d, *J*_trans_ = 16.0 Hz, H_a_), 7.33 (2H, t, *J* 7.5 Hz, 3′,5′-H), 7.40–7.447 (3H, m, ArH), 7.65 (2H, d, *J = *7.8 Hz, ArH), 7.73 (1H, d, *J* = 7.8 Hz, 8-H), 7.82 (2H, t, *J* = 8.5 Hz, 2′,6′-H), 7.95 (1H, d, *J*_trans_ = 16.0 Hz, H_b_), 8.10 (1H, dd, *J* = 2.5 and 7.8 Hz, 7-H), 8.28 (1H, d, *J* = 2.5 Hz, 5-H), 12.40 (1H, s, NH); δ_C_ (125 MHz, DMSO-*d*_6_) 116.6 (d, ^2^*J*_CF_ = 21.75 Hz), 121.1, 121.4, 128.6, 128.8 (d, ^3^*J*_CF_ = 8.0 Hz), 129.0, 129.7, 131.3, 131.4, 131.9 (d, ^4^*J*_CF_ = 2.8 Hz), 132.8, 134.9, 135.3, 136.7, 138.3, 151.5, 161.7, 162.0 (d, ^1^*J*_CF_ = 243.6 Hz); *m*/*z* 343 (100, MH^+^); HRMS (ES): MH^+^. found 343.1247. C_22_H_16_N_2_OF^+^ requires 343.1238.

*(E)-6-(4-Fluorophenyl)-2-(4-fluorostyryl)quinazolin-4(3H)-one* (**6f**). Solid (0.20 g, 63%), mp. 345 °C; ν_max_ (ATR) 508, 551, 823, 968, 1157, 1237, 1476, 1659, 3174 cm^−1^; δ_H_ (500 MHz, DMSO-*d*_6_) 6.96 (1H, d, *J*_trans_ = 16.0 Hz, H_a_), 7.29 (2H, t, *J* = 9.0 Hz, 3′,5′-H), 7.32 (2H, t, *J* = 9.0 Hz, 3″,5″-H), 7.72 (2H, t, *J* = 9.0 Hz, 2″,6″-H), 7.73 (1H, d, *J* 8.5 Hz, 8-H), 7.81 (2H, t, *J* = 9.0 Hz, 2',6'-H), 7.94 (1H, d, *J*_trans_ = 16.0 Hz, H_b_), 8.10 (1H, dd, *J* = 2.0 and 8.5 Hz, 7-H), 8.29 (1H, d, *J* = 2.0 Hz, 5-H), 12.40 (1H, br s, NH); δ_C_ (125 MHz, DMSO-*d*_6_) 116.4 (d, ^2^*J*_CF_ = 21.7 Hz), 121.9, 122.3, 123.6, 128.3, 128.7, 129.3 (d, ^3^*J*_CF_ = 8.5 Hz),129.6, 129.7, 133.5, 134.4, 134.6, 135.7 (d, ^4^*J*_CF_ = 2.8 Hz), 137.3, 137.4, 152.6, 161.6, 163.1 (d, ^1^*J*_CF_ = 243.0 Hz); *m*/*z* 361 (100, MH^+^); HRMS (ES): MH^+^ found 361.1143. C_22_H_15_N_2_OF_2_^+^ requires 361.1152.

*(E)-2-(4-Chlorophenyl)-6-(4-fluorostyryl)quinazolin-4(3H)-one* (**6g**). Solid (0.20 g, 65%), mp. 345 °C; ν_max_ (ATR) 494, 523, 811, 967. 1092, 1241, 1474, 1490, 1518, 1665, 3173 cm^−1^; δ_H_ (500 MHz, DMSO-*d*_6_) 7.01 (1H, d, *J*_trans_ = 16.0 Hz, H_a_), 7.32 (2H, d, *J* = 9.0 Hz, 3″,5″-H-), 7.51 (2H, t, *J* 8.5 Hz, 3,5-H), 7.68 (2H, d, *J* = 8.5 Hz, 2″,6″-H), 7.73 (1H, d, *J* = 8.5 Hz, 8-H), 7.81 (2H, d, *J* = 9.0 Hz, 2,6-H), 7.93 (1H, d, *J*_trans_ = 16.0 Hz, H_b_), 8.10 (1H, dd, *J* = 2.0 and 8.5 Hz, 7-H), 8.29 (1H, d, *J* = 2.0 Hz, 5-H), 12.4 (1H, s, NH); *m*/*z* 377 (100, MH^+^); HRMS (ES): MH^+^ found 377.0852. C_22_H_15_N_2_O^35^ClF requires 377.0857.

*(E)-6-(4-Fluorophenyl)-2-(3-methoxystyryl)quinazolin-4(3H)-one* (**6h**). Solid (0.22 g, 72%), mp. 315 °C; ν_max_ (ATR) 493, 672, 762, 841, 1249, 1433, 1669 cm^−1^; δ_H_ (500 MHz, DMSO-*d*_6_) 3.80 (3H, s, -OCH_3_), 6.98 (1H, dd, *J* 2.0 and 9.0 Hz, 6′-H), 7.02 (1H, d, *J*_trans_ = 16.0 Hz, H_a_), 7.21 (1H, d, *J* = 2.0 Hz, 2′-H), 7.22 (1H, d, *J* = 7.5 Hz, 4′-H), 7.32 (2H, t, *J* = 7.8 Hz, 3,5-H), 7.36 (1H, t, *J* = 7.5 Hz, 5′-H), 7.73 (1H, d, *J* = 8.5 Hz, 8-H), 7.81 (2H, t, *J* = 7.5 Hz, 2,6-H), 7.92 (1H, d, *J*_trans_ = 16.0 Hz, H_b_), 8.10 (1H, dd, *J* = 2.0 and 8.5 Hz, 7-H), 8.29 (1H, d, *J* = 2.0 Hz, 5-H), 12.37 (1H, s, NH); δ_C_ (125 MHz, DMSO-*d*_6_) 55.6, 113.1, 116.1, 116.4 (d, ^2^*J*_CF_ = 20.9 Hz), 120.5, 121.8, 121.9, 123.6, 128.3, 129.3 (d, ^3^*J*_CF_ = 7.5 Hz), 130.6, 133.4, 135.3 (d, ^4^*J*_CF_ = 2.9 Hz), 136.7, 137.3, 137.8, 148.7, 151.9, 160.1, 162.1, 162.6 (d, ^1^*J*_CF_ = 243.7 Hz); *m*/*z* 373 (100, MH^+^); HRMS (ES): MH^+^ found 373.1346. C_23_H_18_N_2_O_2_F^+^ requires 373.1352.

### 3.5. Materials and Methods for Bioassays

*Cytotoxicity studies* [25]: The SRB Assay was performed at the Council for Scientific Research (CSIR, Pretoria, South Africa) in accordance with the protocol of the Drug Evaluation Branch of the National Cancer Institute (NCI, Rockville, MD, USA) and the assay has been adopted for this screening. The human cell lines TK-10, UACC-62 and MCF-7 were obtained from NCI in the framework a collaborative research program between CSIR and NCI. Cell lines were routinely maintained as monolayer cell cultures at 37 °C, 5% CO_2_, 95% air and 100% relative humidity in RPMI containing 5% fetal bovine serum, 2 mM l-glutamine and 50 µg/mL gentamicin. For screening experiment, the cells (3–19 passages) were inoculated in a 96-well microtiter plates at plating densities of 7–10,000 cells/well and were incubated for 24 h. After 24 h the cells were treated with the experimental drugs which were previously dissolved in DMSO and diluted in medium to produce 5 concentrations: 100–0.01 µM (5 × 10-fold serial dilutions). Cells without drug addition served as control and the blank contained complete medium without cells with parthenolide as a standard drug at concentrations: 100–0.01 µM (5 × 10-fold serial dilutions). The plates were incubated for 48 h after addition of the compounds. Viable cells were fixed to the bottom of each well with cold 50% trichloroacetic acid, washed, dried and dyed by SRB. Unbound dye was removed and protein-bound dye was extracted with 10 mM Tris base for optical density determination at the wavelength 540 nm using a multi-well spectrophotometer. Data analysis was performed using GraphPad Prism software. 50% of cell growth inhibition (IC_50_) was determined by linear regression.

*In vitro antibacterial microdilution assay*: The microplate serial dilution assay developed by Eloff [27] was used to determine the minimal inhibitory concentration of the compounds against six Gram-positive and four Gram-negative bacterial strains (Table 5). The microbial test organisms were prepared using the method described by Pauw and Eloff [26]. The bacterial cultures were maintained on Müller-Hilton (MH) agar at 4 °C and in Müller-Hilton broth at 37 °C prior to use for the determination of minimal inhibitory concentrations. Three to five colonies of bacteria from fresh 24 h agar plate culture were inoculated into fresh MH broth and incubated for 14 h prior to antibacterial testing. This experiment was performed in triplicate and repeated twice for verification. Each test compound was dissolved in the appropriate solvent (DMSO or acetone) to a concentration of 1 mg/mL and serially diluted two-fold in a 96-well microplate with equal volumes of water for each of the 10 bacteria used. 100 μL of freshly prepared bacterial cultures of each bacterium were added to each well. DMSO was used as negative control and gentamicin as the positive control to confirm the sensitivity of the system. The plates were then incubated overnight at 37 °C in 100% relative humidity. As an indicator of bacterial growth, 40 μL of 0.2 mg/mL INT was added to each well after incubation and the plates incubated further at 37 °C for 2 h. The MIC was determined and recorded as the lowest concentration of the compounds at which the bacterial growth was inhibited.

**Table 5:** Bacterial test organisms used in this study.

Bacteria	Strain
*Escherichia coli*	ATCC 35218, Gram-negative
*Pseudomonas aeruginosa*	ATCC 7700, Gram-negative
*Salmonella typhi*	ATCC 14028, Gram-negative
*Acinetobacter calcaoceuticals anitratus*	CSIR isolate, Gram-negative
*Bacillus cereus*	ATCC 10702, Gram-positive
*Enterococcus hirae*	ATCC 8043, Gram-positive
*Enterococcus faecalis*	ATCC 19433, Gram-positive
*Enterococcus gallinarium*	ATCC 49573, Gram-positive
*Enterococcus casseliflavus*	ATCC 25788, Gram-positive
*Staphylococcus aureus*	ATCC 25925, Gram-positive

*In vitro antifungal microdilution assay*: For the antifungal assay, the microplate serial dilution assay developed by Eloff [26] and modified by Masoko *et al.* [28] was used. The fungal strains (Table 6) were maintained on Sabouraud dextrose agar and Sabouraud dextrose broth prior to inhibition tests. Two-fold serial dilutions of the compounds were prepared as described for the bacteria. Using sterile cotton swabs, actively growing fungal organisms were transferred from agar to Sabouraud dextrose broth and 100 μL of the cultures was then added to each well as described for the bacteria in the previous section. DMSO was used as negative control and Amphotericin-B as the positive control. As an indicator of fungal growth, 40 μL of 0.2 mg/mL INT was added to each well and the plates were incubated at 30 °C after which the MIC was determined after 24 h incubation.

**Table 6:** Fungal test organisms used in this study.

Fungi	Strain
*Candida albicans*	ATCC 24433, yeast
*Cryptococcus neoformans*	ATCC 14116, yeast

### 3.6. Molecular Docking Studies

*Protein Structure*: The starting structures of human dihydrofolate reductase (DHFR) and thymidylate synthase (TS) enzyme were obtained from PDB with id 3GHW^201^ and 1I00 [12], respectively. All water molecules and inhibitors were removed except the co-factor NADPH in DHFR and dUMP in TS remained as part of the protein. Polar hydrogen atoms, Kollman-Amber united atom partial charges and solvation parameters were added by utilizing AutoDockTools [13].

*Compound Structure*: The initial coordinates for compounds **5a**, **b**, **d**, and **6a**, **b**, **d**–**f** were generated using Hyperchem 7.0 (Hypercube Inc., Gainesville, FL, USA). The initial coordinates of inhibitor for DHFR (*N*-{4-[(2,4-diamino-5-ethyl-7*H*-pyrrolo[2,3-*d*]pyrimidin-6-yl)sulfanyl]benzoyl}-l-glutamic acid) and TS (Tomudex) were obtained from the ligand of PDB id 3GHW and 100, respectively, and used as control docking. All compounds/inhibitors were retained with only polar hydrogen atoms. Gasteiger charges and torsional angles were added by utilizing AutoDockTools.

*Molecular Docking Simulation*: Grid maps of 60 × 60 × 60 points with 0.375 Å spacing were centered at the ligand binding site in the crystal structures. The docking was performed employing Lamarckian genetic algorithm with pseudo-Solis and Wets local search with population size of 150 and energy evaluation of 2500,000; root mean square tolerance of 1.0 Å for control docking and 2.0 Å for the test compounds; and 200 docking runs by AutoDock 4.2 [32]. The ligand conformation with lowest free energy of binding in the most populated cluster was selected for comparison.

## 4. Conclusions

The 6-bromo-2-styrylquinazolin-4(3*H*)-ones are suitable candidates for palladium-catalyzed Suzuki-Miyaura cross-coupling to afford 6-aryl–substituted 2-styrylquinazolinones with potential biological activity. In general, the 6-bromo-2-styrylquinazolinon-4-ones were found to exhibit more significant inhibitory activity than the corresponding 6-aryl–substituted derivatives. However, the presence of a 4-chlorostyryl group at the 2-positions of the 6-bromoquinazolin-4(3*H*)-one framework is not favourable for cytotoxicity. A combination of a substituted benzene ring at the 2- and 6-position resulted in diminished activity against the cancer cell lines, as observed for 6-(4-fluorophenyl)–substituted derivatives **6g** and **6h** bearing 4-chlorostyryl and 3-methoxystyryl groups, respectively. Some of the compounds with high cytotoxicity against cancer cells (*i.e.*, **5a**–**d**, **6a** and **6b**) were also found to exhibit good antibacterial and antifungal activity. Overall, the test compounds had moderate bacteriostatic and fungistatic activity. The observed results serve as a guide for the design and synthesis of more potent styrylquinazolinones with dual anticancer and antimicrobial activity to inhibit bacterial and fungal infections in frequently immune compromised cancer patients. Structure-activity relationship and molecular docking indicate that the entire 2-styrylquinazolinone framework is required for biological activity, however, activity is enhanced by a halide or small lipophilic group at the 6-position of the scaffold.

## Supplementary Materials

Supplementary materials can be accessed at: http://www.mdpi.com/1420-3049/21/1/28/s1. Copies of ^1^H- and ^13^C-NMR spectra of **2**, **3**a–**d** and **4a**–**h** (S1) and the % cell viability and IC_50_ values of parthenolide and compounds **3a**–**d** and **4a**–**h** (S2).
