# Supplementary material for: Synthesis, Biological Evaluation and Molecular Docking Studies of 6-Aryl-2-Styrylquinazolin-4(3H)-Ones
Source: Molecules. 2015 Dec 25;21(1):28. doi: 10.3390/molecules21010028 (PMC6274206; doi:10.3390/molecules21010028)
Supplement: Supplementary file 1 [file molecules-21-00028-s001.pdf]

Supplementary Materials: Synthesis, Biological Evaluation and Molecular Docking Studies of the 6-Aryl-2-Styrylquinazolin-4(3*H*)-Ones

Emmanuel Ndubuisi Agbo [1] Tshepiso Jan Makhafola [2] Yee Siew Choong [3], Malose Jack Mphahlele [1,*] and Ponnadurai Ramasami [4]

S1. Copies of ^{1}H- and ^{13}C-NMR Spectra for Compounds 4, 5a–d and 6a–h

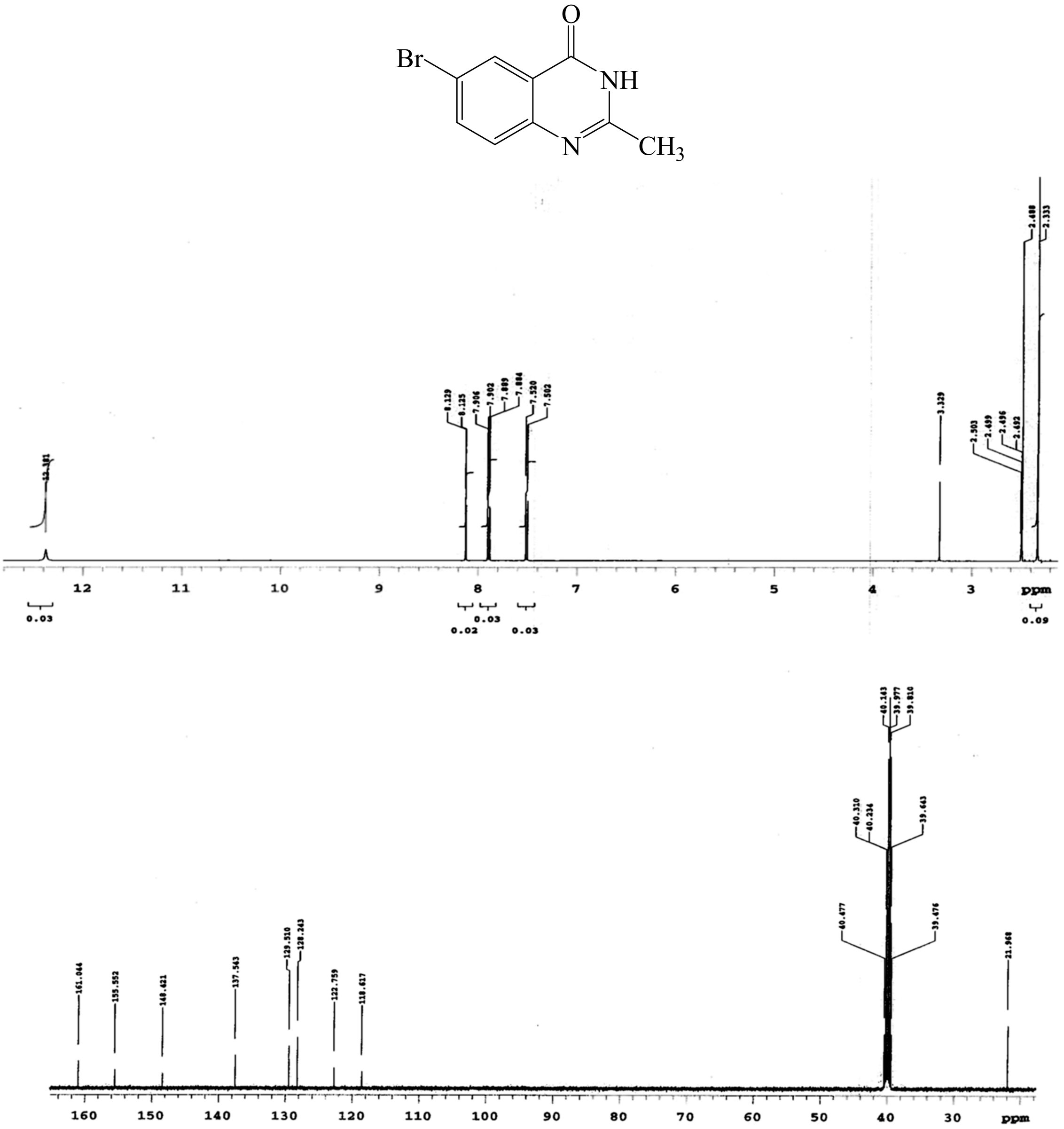

Figure S1. ^{1}H- and ^{13}C-NMR spectra of compound **4** in DMSO-d_6.

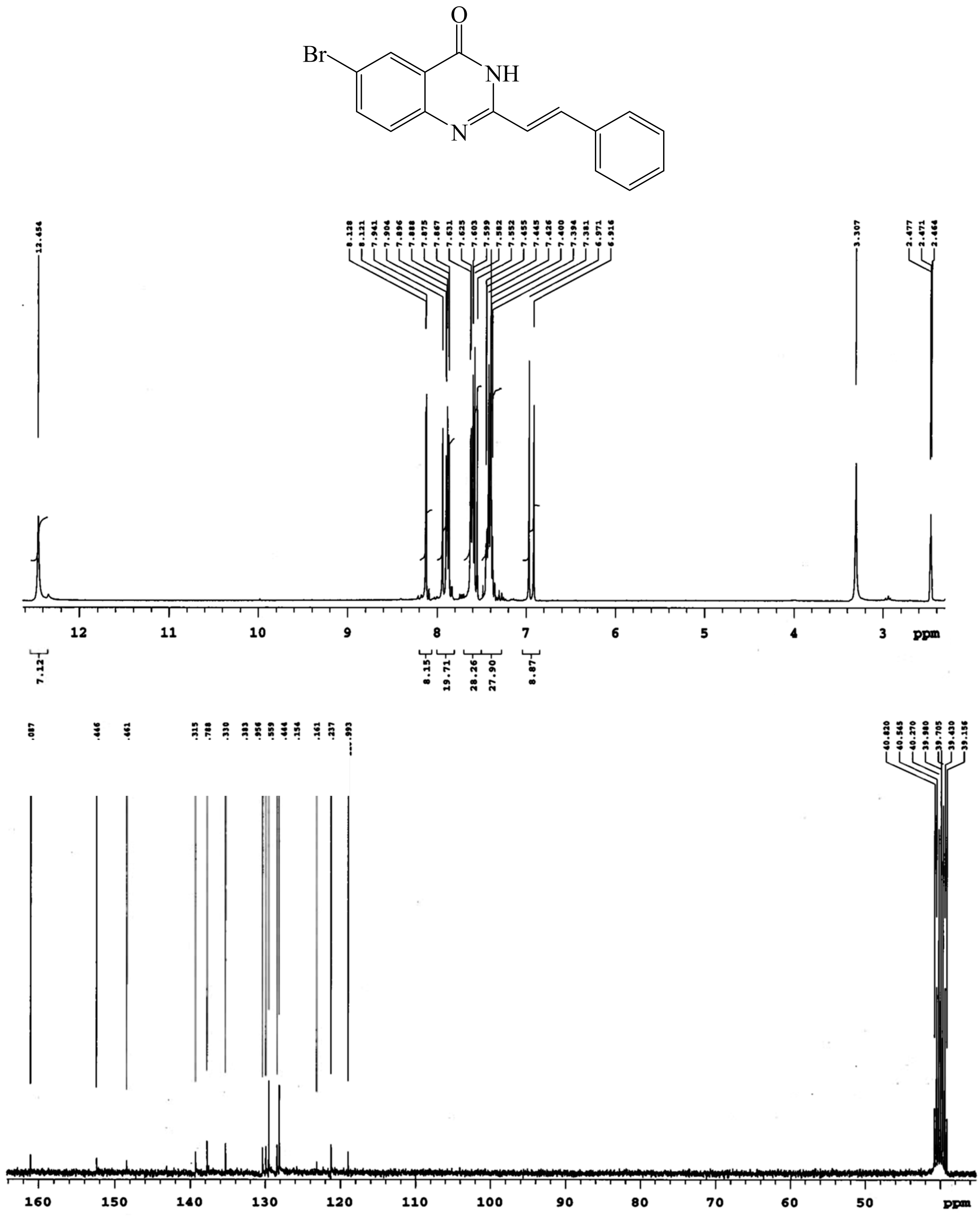

Figure S2. ^{1}H- and ^{13}C-NMR spectra of compound **5a** in DMSO-d_6.

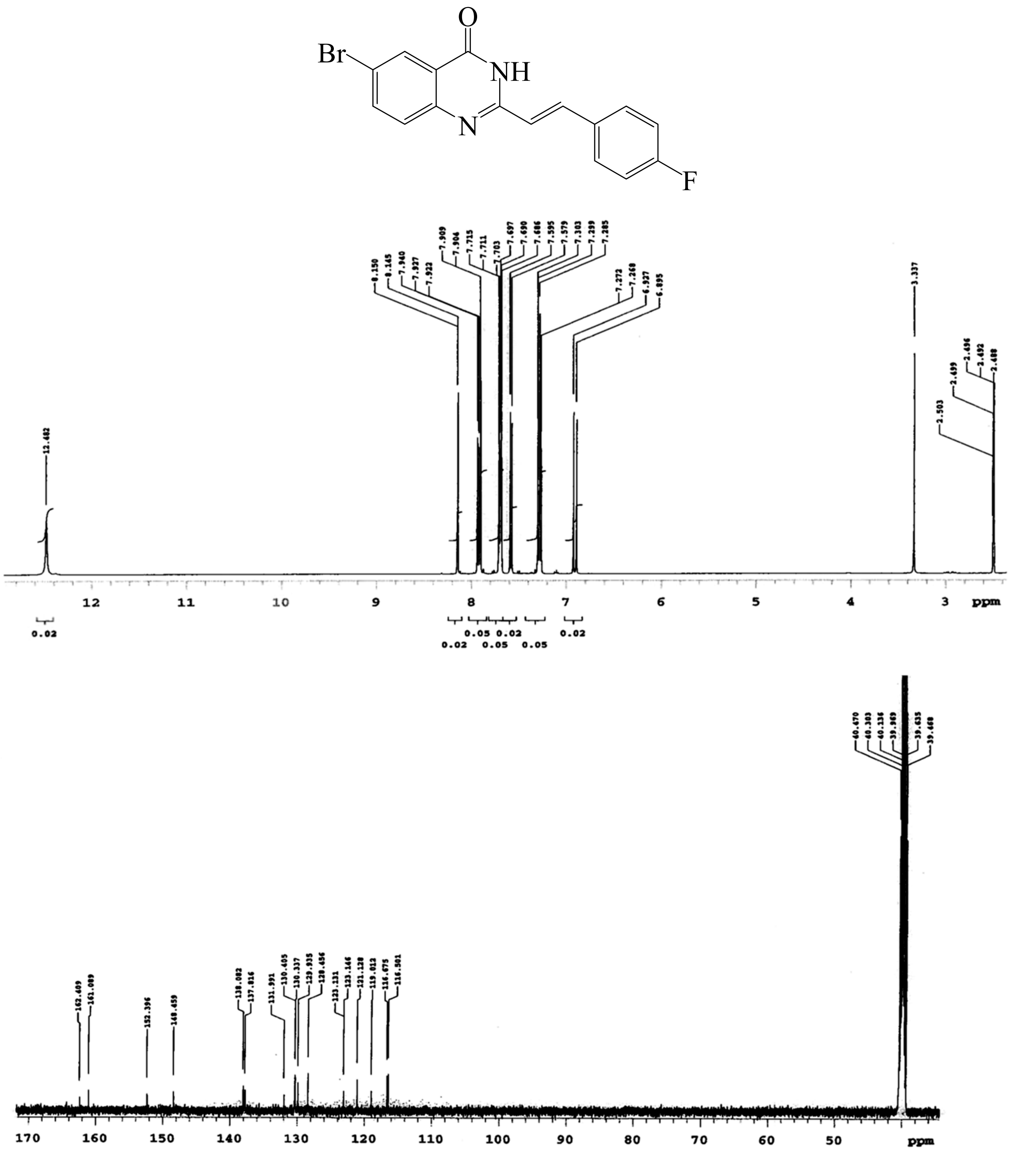

Figure S3. ^{1}H- and ^{13}C-NMR spectra of compound **5b** in DMSO-d_6.

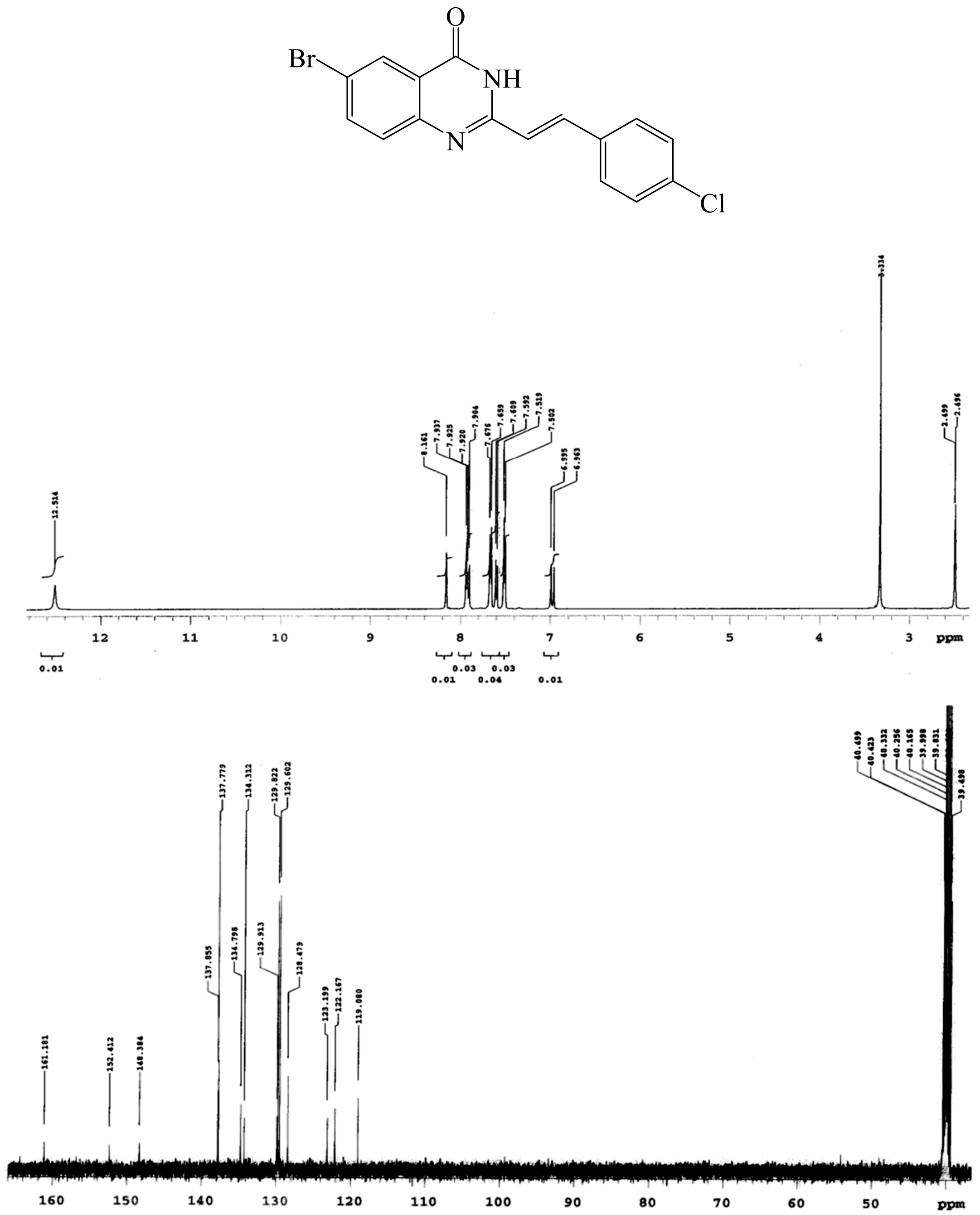

Figure S4. ^{1}H- and ^{13}C-NMR spectra of compound **5c** in DMSO-d_6.

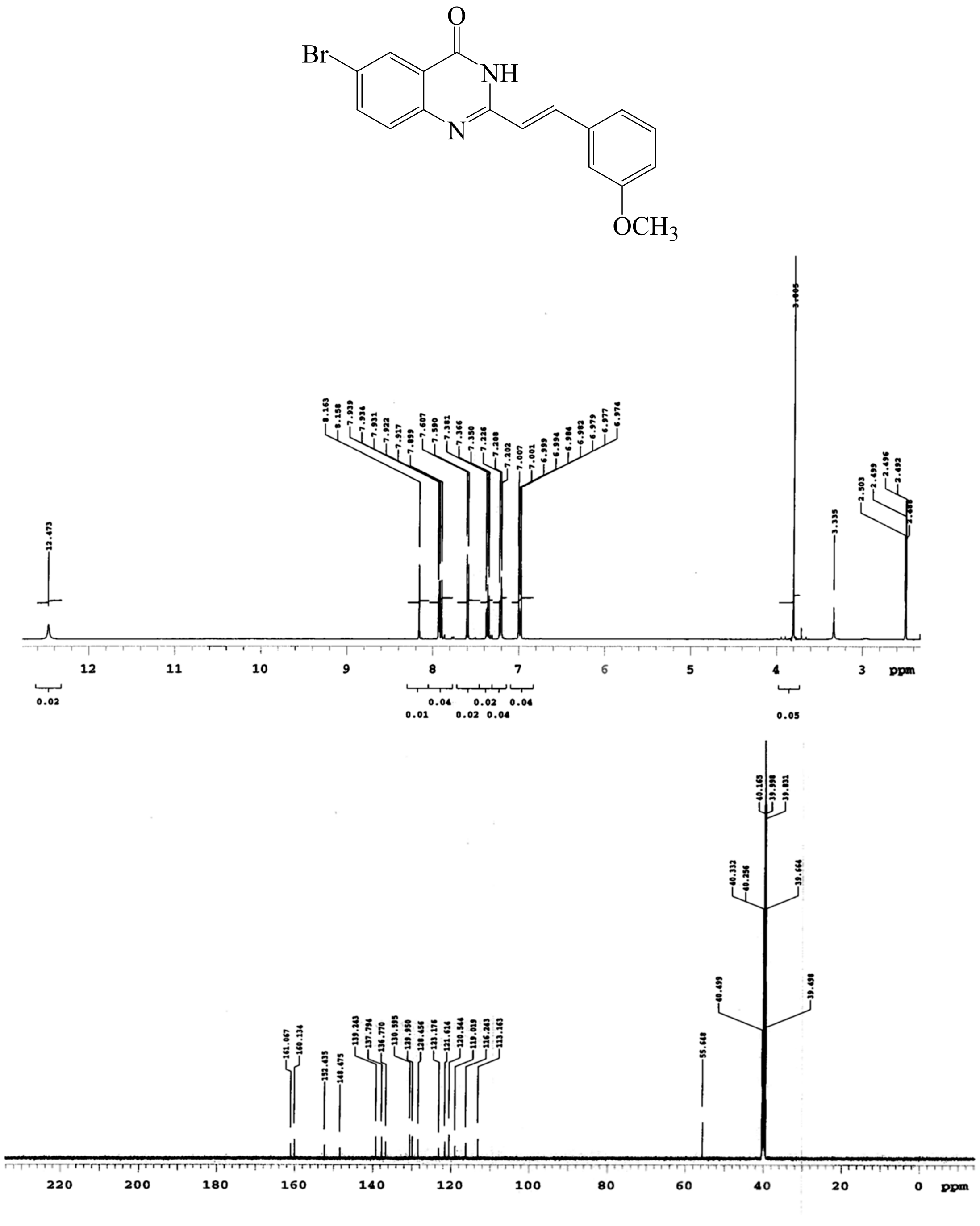

Figure S5. ^{1}H- and ^{13}C-NMR spectra of compound **5d** in DMSO-d_6.

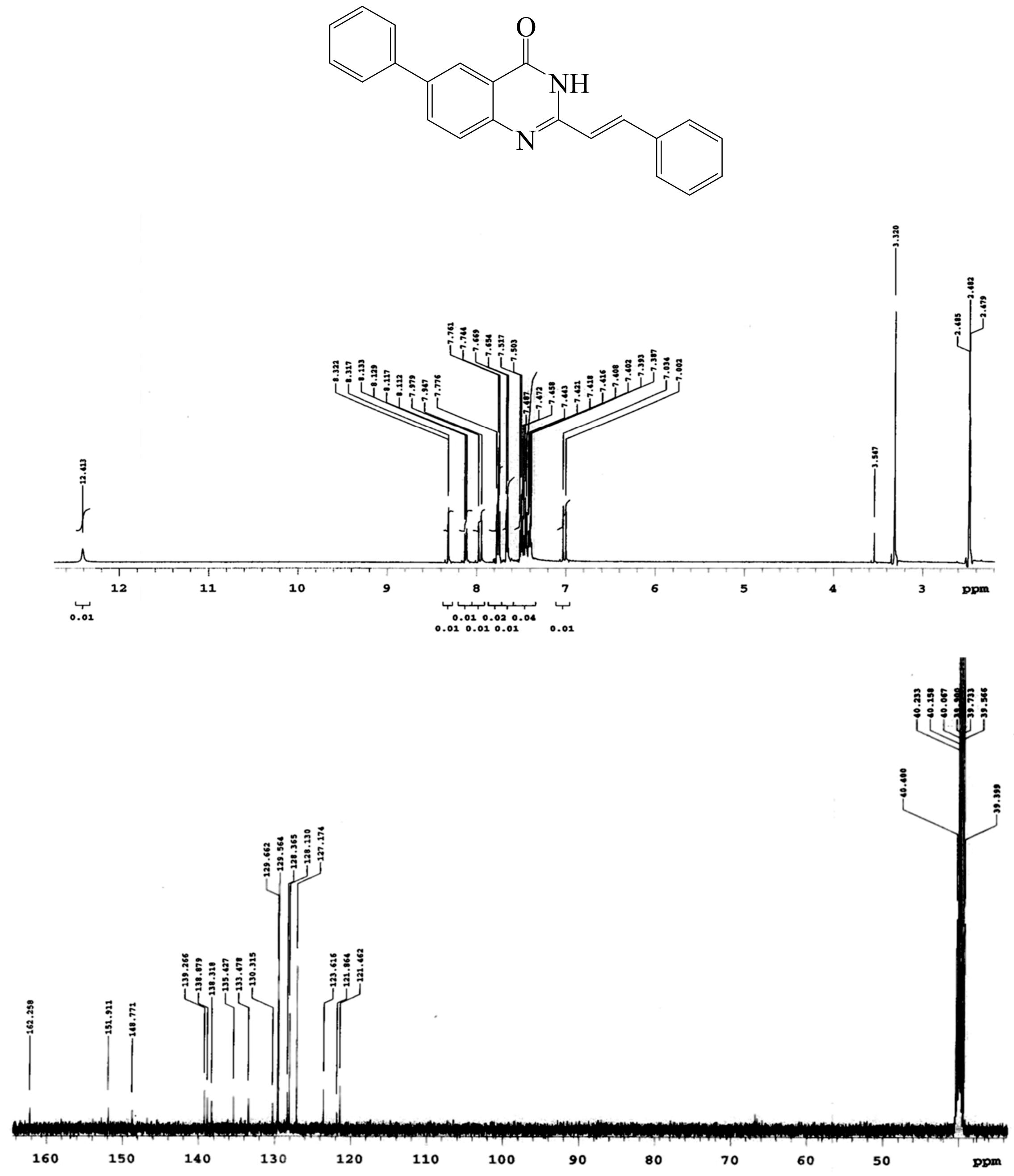

Figure S6. ^{1}H- and ^{13}C-NMR spectra of compound **6a** in DMSO-d_6.

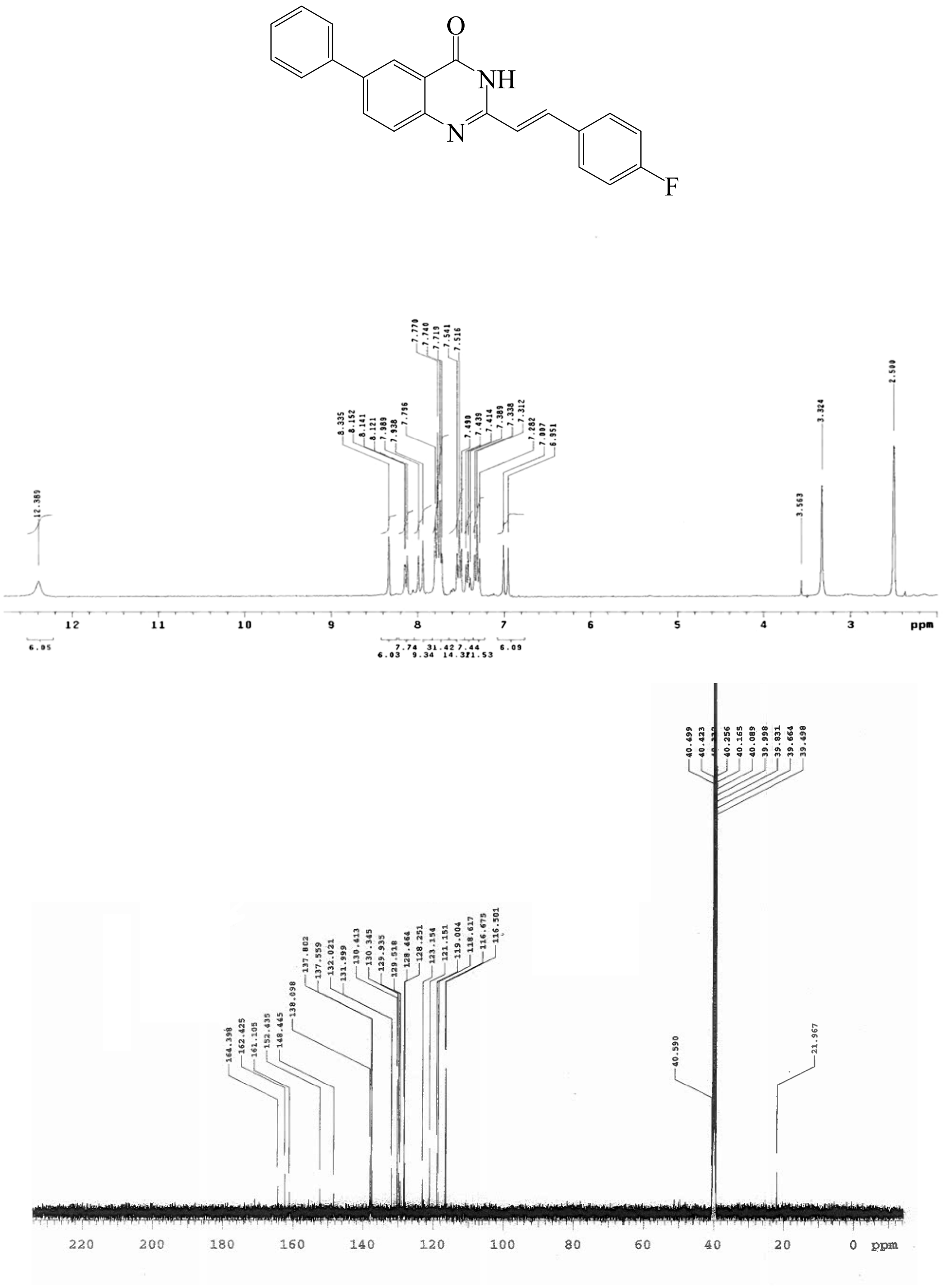

Figure S7. ^{1}H- and ^{13}C-NMR spectra of compound **6b** in DMSO-d_6.

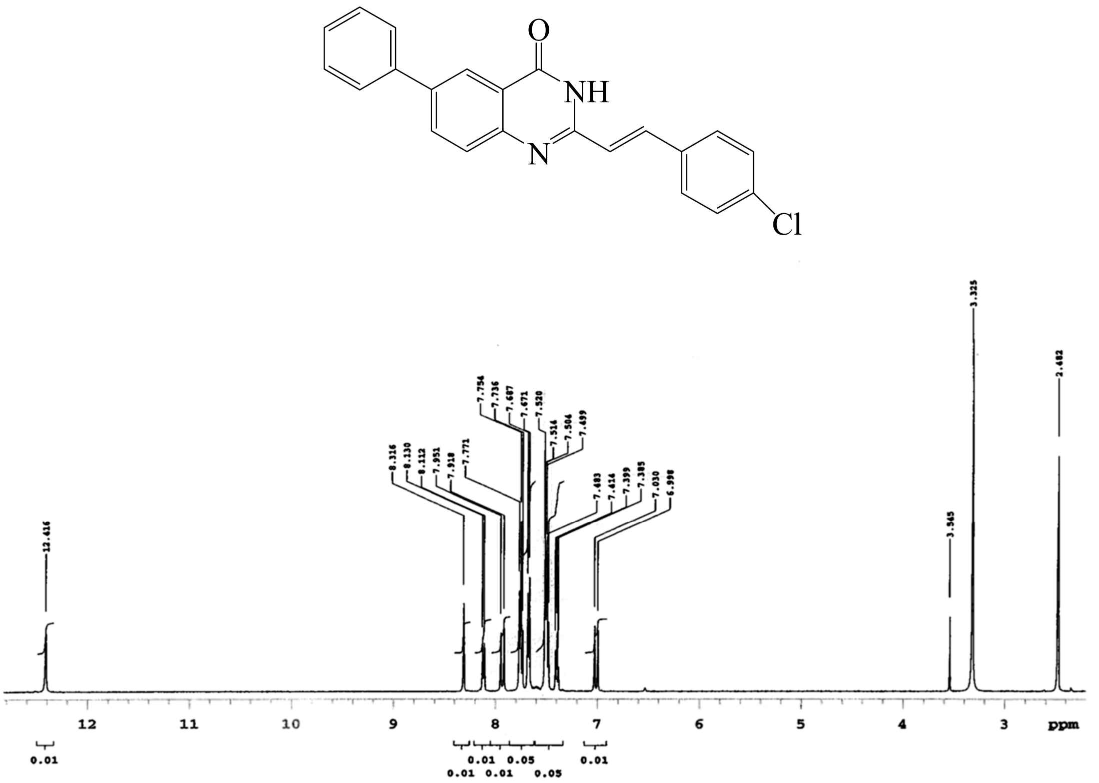

Figure S8. ^{1}H-NMR spectrum of compound **6c** in DMSO-*d*$_6$.

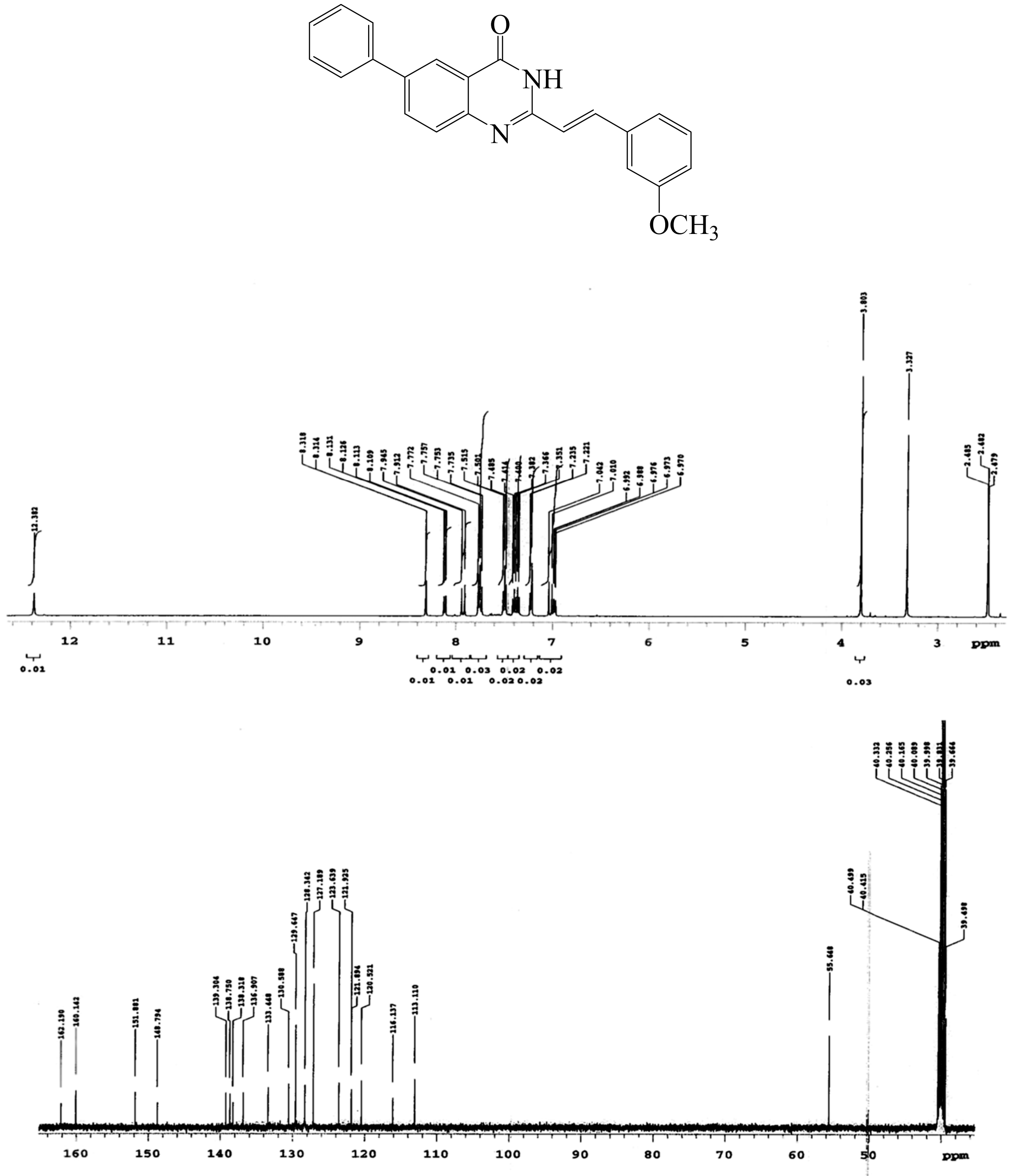

Figure S9. ¹H- and ¹³C-NMR spectra of compound **6d** in DMSO-*d*₆.

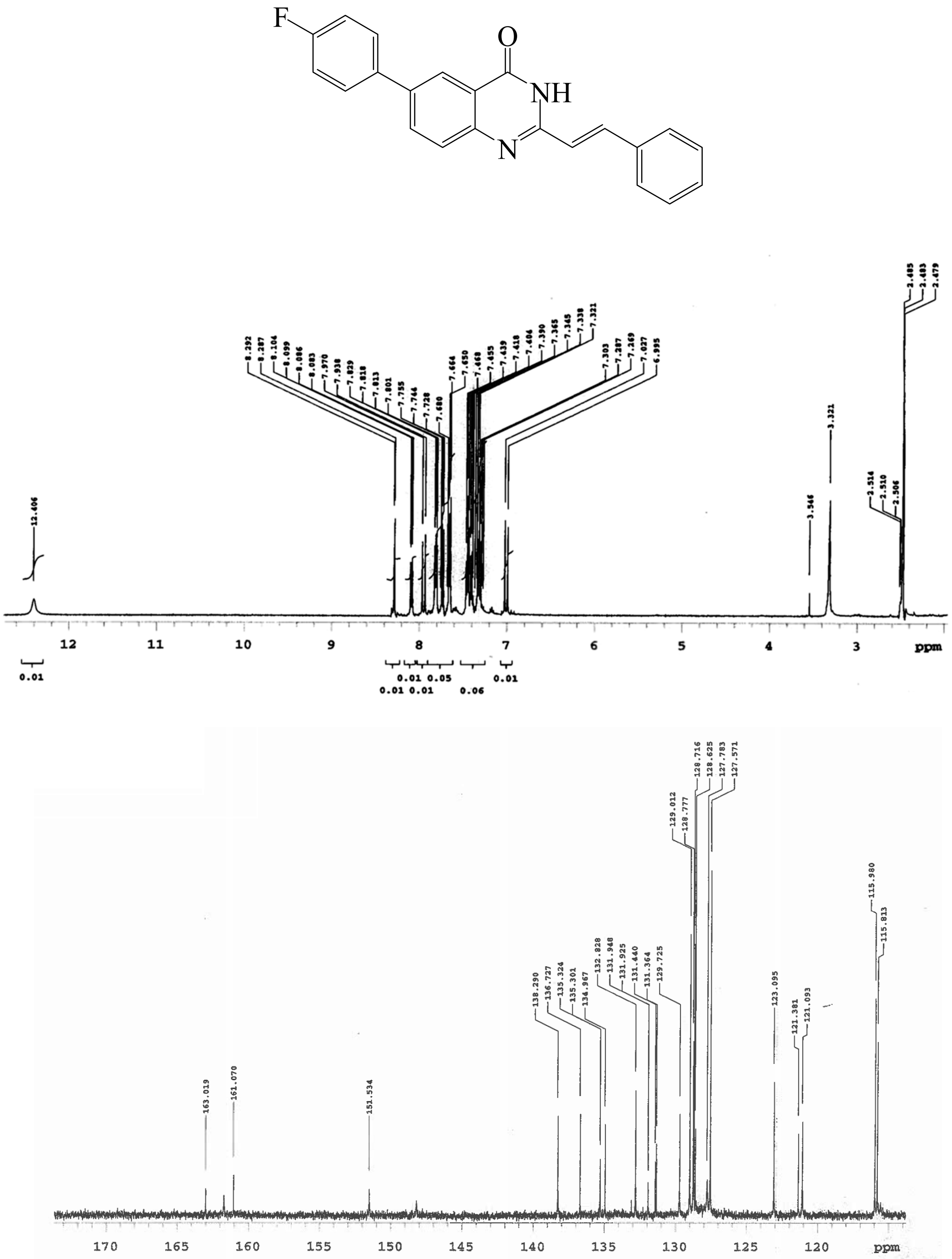

Figure S10. ^{1}H-NMR and enlarged portion of ^{13}C-NMR spectra of compound **6e** in DMSO-d_6.

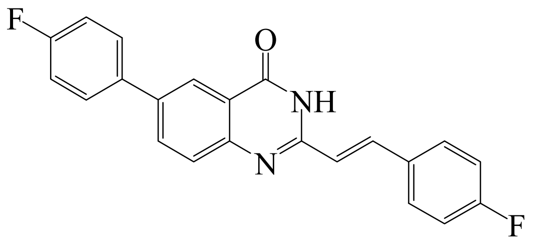

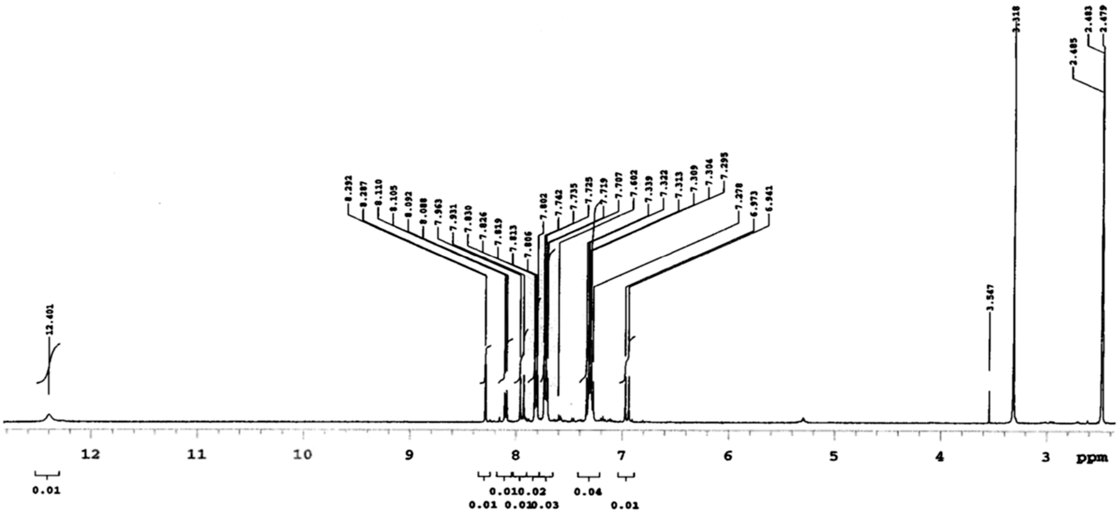

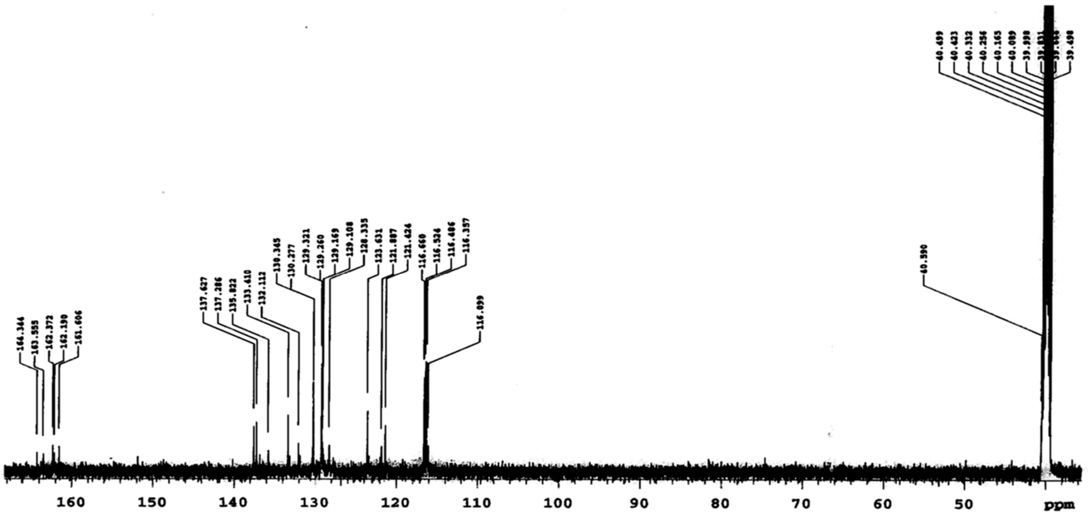

Figure S11. ^{1}H- and ^{13}C-NMR spectra of compound **6f** in DMSO-d_6.

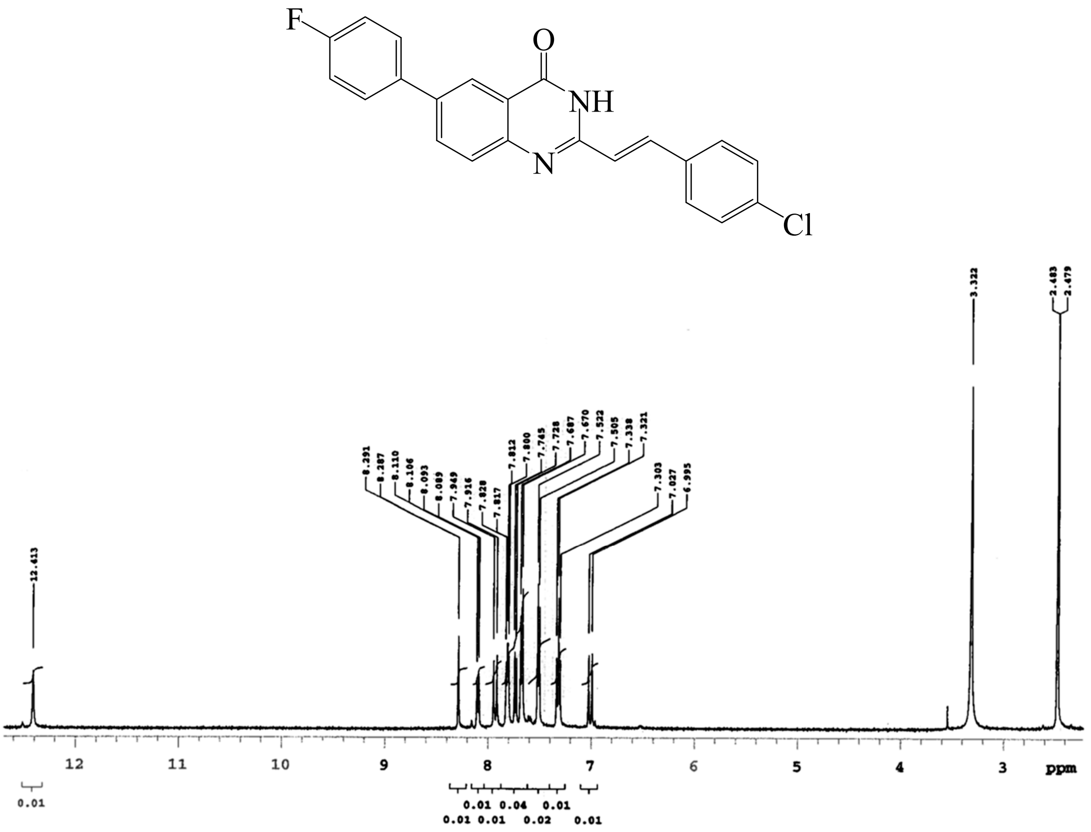

Figure S12. ^{1}H-NMR spectrum of compound 6**g** in DMSO-*d*6.

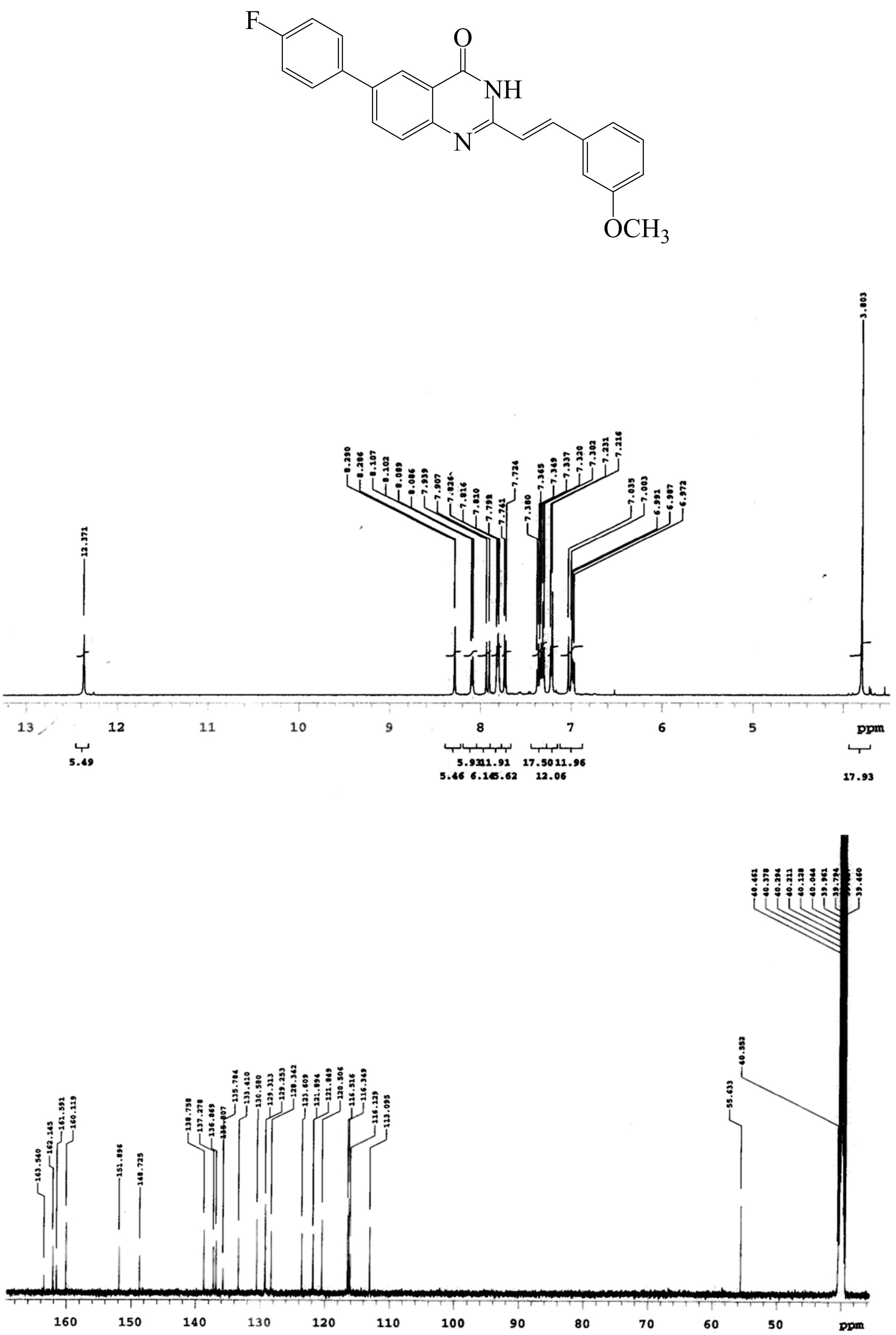

Figure S13. ^{1}H- and ^{13}C-NMR spectra of compound **6h** in DMSO-d_6.

S2. % Cell Viability of TK-10, UACC-62 and MCF-7 cells Exposed Parthenolide, 5a–d and 6a–h

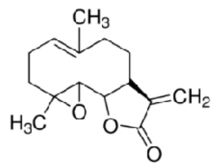

Parthenolide

Table S1. Percentage cell viability of TK-10, UACC-62 and MCF-7 cells exposed to different concentrations of **parthenolide**.

Conc. (μM)	Log Conc.	%Viability TK-10	SD	%Viability UACC-62	SD	%Viability MCF-7	SD
100	2	3.61	0.24	3.30	0.08	15.00	0.45
10	1	15.06	0.50	55.35	3.08	33.02	1.01
1	0	93.55	4.15	98.84	9.97	67.70	1.29
0.1	−1	100	4.05	100	7.93	99.05	0.54
0.01	−2	100	4.03	100	9.05	100	2.56

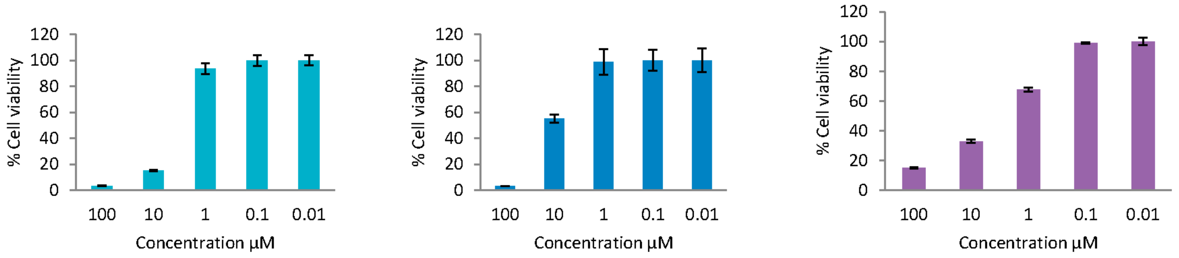

TK-10 UACC-62 MCF-7

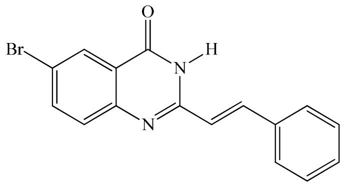

5a

Table S2. Percentage cell viability of TK-10, UACC-62 and MCF-7 cells exposed to different concentrations of **5a**.

Conc. (μM)	Log Conc.	%Viability TK-10	SD	%Viability UACC-62	SD	%Viability MCF-7	SD
100	2	13.58	0.49	18.99	0.12	32.49	2.27
10	1	51.07	2.47	24.96	0.41	34.34	0.95
1	0	79.73	1.08	46.02	0.17	56.35	0.02
0.1	−1	96.69	0.96	95.73	3.91	69.93	0.45
0.01	−2	100	1.39	98.10	1.33	77.30	0.49

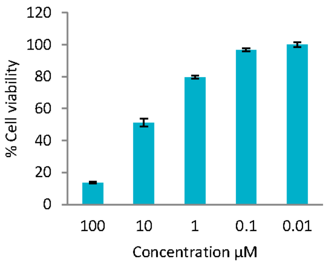

TK-10

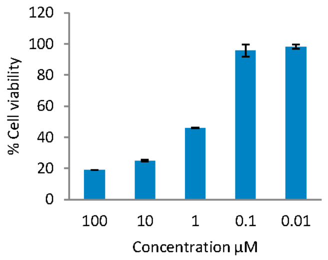

UACC-62

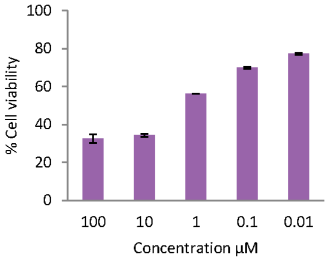

MCF-7

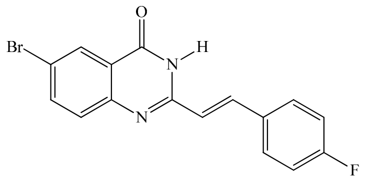

5b

Table S3. Percentage cell viability of TK-10, UACC-62 and MCF-7 cells exposed to different concentrations of **5b**.

Conc. (µM)	Log Conc.	%Viability TK-10	SD	%Viability UACC-62	SD	%Viability MCF-7	SD
100	2	14.68	0.16	6.82	0.04	21.83	0.08
10	1	35.80	0.94	37.82	4.38	48.26	0.86
1	0	62.63	1.42	49.52	0.24	59.53	0.91
0.1	−1	75.92	1.74	63.03	0.67	68.43	0.28
0.01	−2	100	0.18	93.98	3.06	99.26	0.04

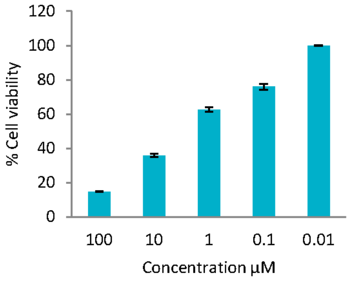

TK-10

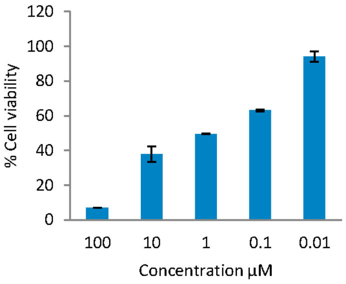

UACC-62

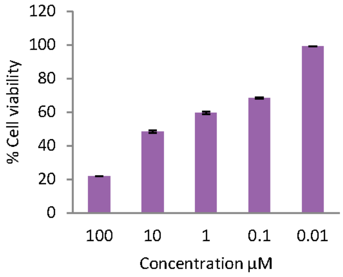

MCF-7

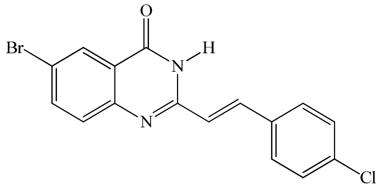

5c

Table S4. **Percentage cell viability of TK-10, UACC-62 and MCF-7 cells exposed to different concentrations of** 5c**.**

Conc. (μM)	Log Conc.	%Viability TK-10	SD	%Viability UACC-62	SD	%Viability MCF-7	SD
100	2	35.35	0.30	35.36	0.31	42.48	1.33
10	1	53.553	0.96	73.55	2.97	96.49	0.11
1	0	83.17	0.66	90.53	9.75	99.92	0.83
0.1	−1	92.99	0.98	95.53	2.86	99.53	0.01
0.01	−2	100	2.54	99.60	1.45	100	0.86

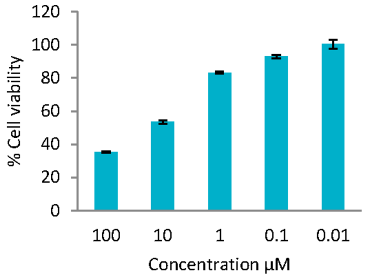

TK-10

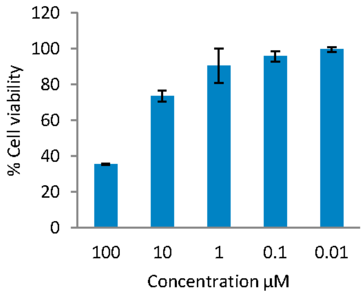

UACC-62

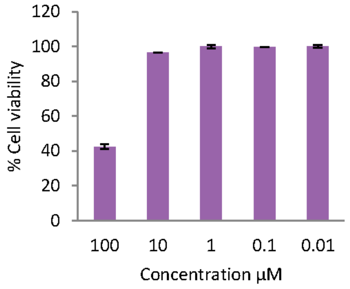

MCF-7

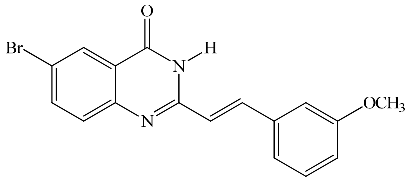

5d

Table S5. Percentage cell viability of TK-10, UACC-62 and MCF-7 cells exposed to different concentrations of **5d**.

Conc. (μM)	Log Conc.	%Viability TK-10	SD	%Viability UACC-62	SD	%Viability MCF-7	SD
100	2	5.10	0.72	5.78	2.54	14.38	4.43
10	1	5.53	0.21	10.61	0.81	36.04	1.16
1	0	53.22	0.13	52.00	1.23	57.33	1.09
0.1	−1	90.69	0.46	59.34	1.49	68.96	1.41
0.01	−2.	100	1.22	97.91	0.004	98.80	0.26

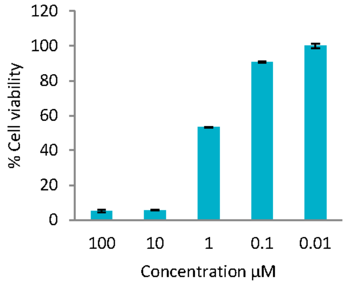

TK-10

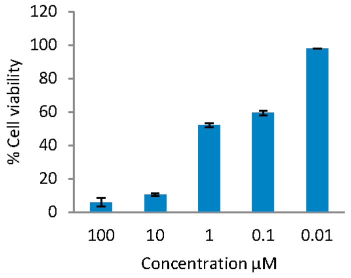

UACC-62

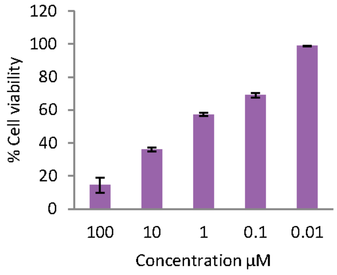

MCF-7

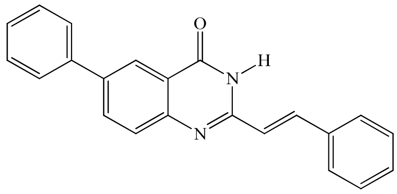

6a

Table S6. Percentage cell viability of TK-10, UACC-62 and MCF-7 cells exposed to different concentrations of **6a**.

Conc. (μM)	Log Conc.	%Viability TK-10	SD	%Viability UACC-62	SD	%Viability MCF-7	SD
100	2	55.00	4.34	40.71	1.44	47.21	0.73
10	1	58.94	3.62	45.27	2.04	54.78	3.20
1	0	71.01	2.33	81.01	1.41	67.71	0.39
0.1	−1	99.81	1.42	97.84	0.31	99.71	0.69
0.01	−2	100	1.63	100	0.29	100	2.41

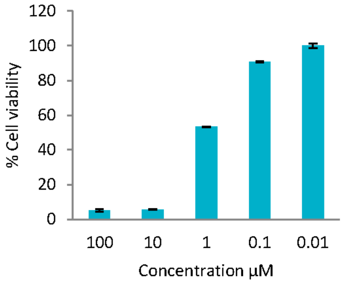

TK-10

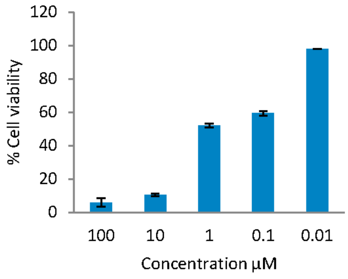

UACC-62

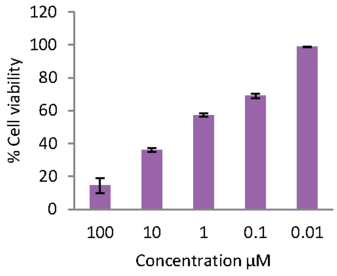

MCF-7

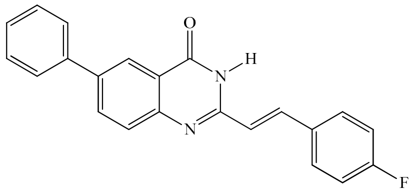

6b

Table S7. Percentage cell viability of TK-10, UACC-62 and MCF-7 cells exposed to different concentrations of **6b**.

Conc. (μM)	Log Conc.	%Viability TK-10	SD	%Viability UACC-62	SD	%Viability MCF-7	SD
100	2	47.67	0.77	45.79	2.51	45.88	1.93
10	1	65.10	0.68	53.95	0.54	52.08	1.65
1	0	99.73	1.28	75.61	0.46	85.19	2.25
0.1	−1	99.50	3.41	92.11	1.53	99.37	0.29
0.01	−2	100	0.73	97.67	2.33	99.74	0.71

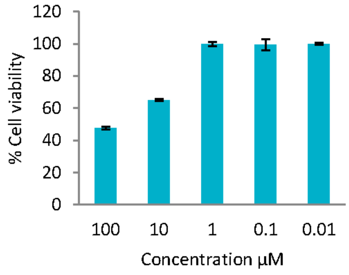

TK-10

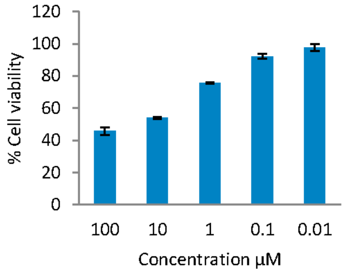

UACC-62

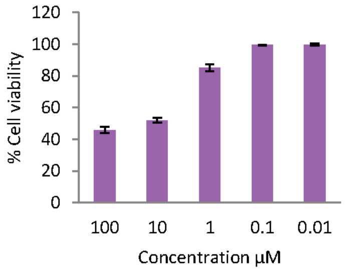

MCF-7

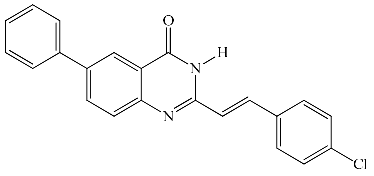

6c

Table S8. Percentage cell viability of TK-10, UACC-62 and MCF-7 cells exposed to different concentrations of **6c**.

Conc. (µM)	Log Conc.	%Viability TK-10	SD	%Viability UACC-62	SD	%Viability MCF-7	SD
100	2	70.55	0.84	63.26	1.46	53.23	2.40
10	1	98.37	2.19	95.40	1.47	95.00	0.27
1	0	100	2.34	99.83	1.09	98.80	0.97
0.1	−1	100	0.28	99.83	1.34	98.60	0.23
0.01	−2.	100	1.87	100	3.60	99.93	0.33

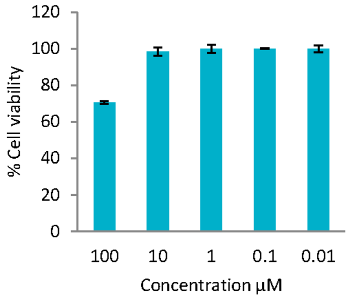

TK-10

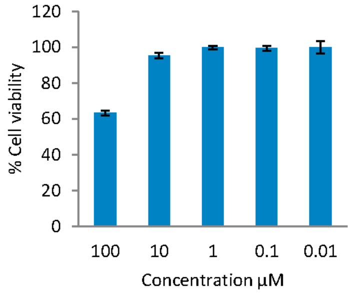

UACC-62

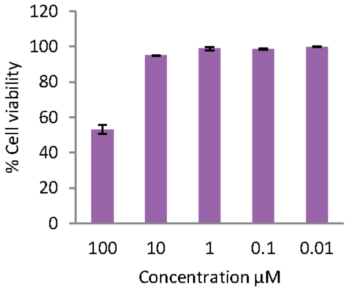

MCF-7

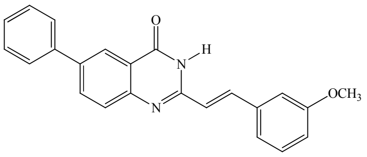

6d

Table S9. Percentage cell viability of TK-10, UACC-62 and MCF-7 cells exposed to different concentrations of **6d**.

Conc. (µM)	Log Conc.	%Viability TK-10	SD	%Viability UACC-62	SD	%Viability MCF-7	SD
100	2	42.85	0.75	33.32	0.71	34.22	0.49
10	1	69.21	1.46	48.30	2.49	58.49	2.70
1	0	95.56	2.35	62.44	0.54	77.81	2.47
0.1	−1	100	2.28	96.86	0.15	99.08	0.52
0.01	−2	100	0.56	100	0.29	98.10	0.57

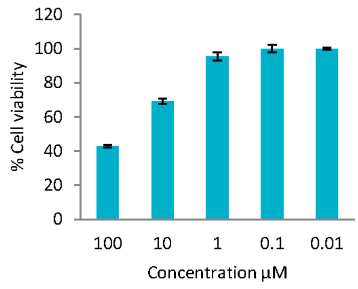

TK-10

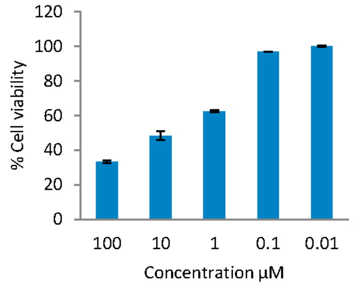

UACC-62

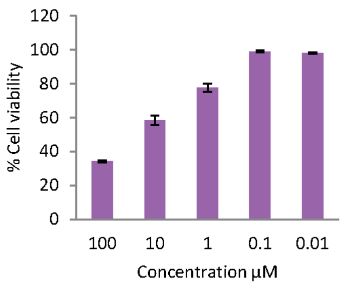

MCF-7

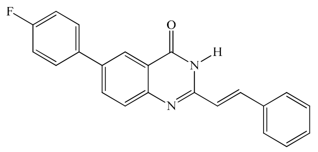

6e

Table S10. Percentage cell viability of TK-10, UACC-62 and MCF-7 cells exposed to different concentrations of **6e**.

Conc. (µM)	Log Conc.	%Viability TK-10	SD	%Viability UACC-62	SD	%Viability MCF-7	SD
100	2	62.72	6.24	50.25	0.93	62.26	3.82
10	1	65.26	0.15	63.82	0.30	46.73	0.66
1	0	90.18	3.81	58.44	0.31	62.15	1.67
0.1	−1	98.30	3.04	95.46	0.16	98.99	0.99
0.01	−2	99.69	2.05	99.87	1.76	99.85	1.24

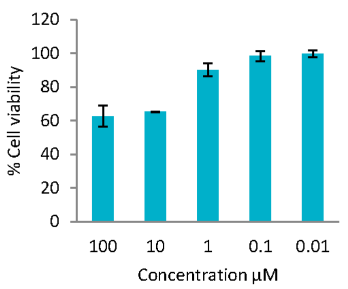

TK-10

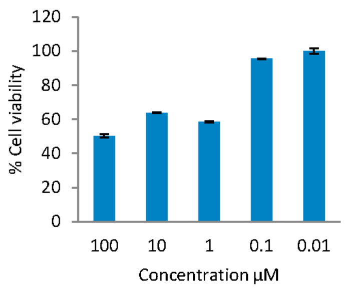

UACC-62

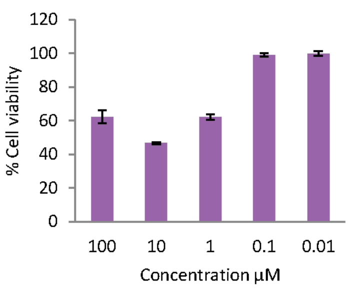

MCF-7

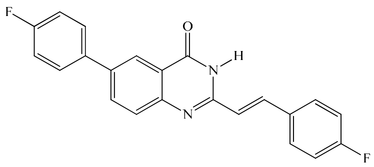

6f

Table S11. Percentage cell viability of TK-10, UACC-62 and MCF-7 cells exposed to different concentrations of **6f**.

Conc. (μM)	Log Conc.	%Viability TK-10	SD	%Viability UACC-62	SD	%Viability MCF-7	SD
100	2	52.86	1.26	38.88	1.40	42.96	2.11
10	1	75.35	2.92	52.22	0.78	46.36	0.30
1	0	100	1.28	71.38	1.15	83.30	1.37
0.1	−1	100	0.08	92.32	1.56	98.95	0.05
0.01	−2	100	2.39	99.79	1.63	99.41	0.20

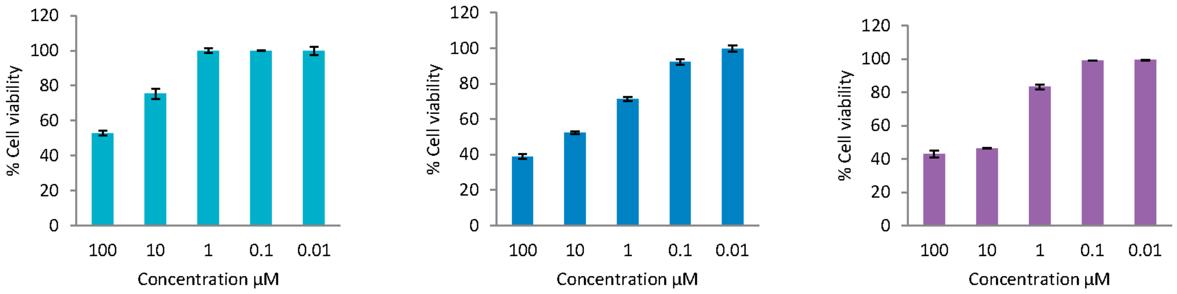

TK-10 **UACC-62** **MCF-7**

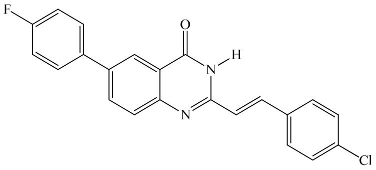

6g

Table S12. Percentage cell viability of TK-10, UACC-62 and MCF-7 cells exposed to different concentrations of **6g**.

Conc. (μM)	Log Conc.	%Viability TK-10	SD	%Viability UACC-62	SD	%Viability MCF-7	SD
100	2	70.77	1.49	65.40	1.49	86.89	3.57
10	1	77.51	2.83	77.87	1.02	97.79	1.07
1	0	98.36	4.58	95.75	0.88	99.62	2.11
0.1	−1	99.18	5.79	99.88	2.23	99.75	0.49
0.01	−2	99.09	2.86	99.46	0.78	100	0.31

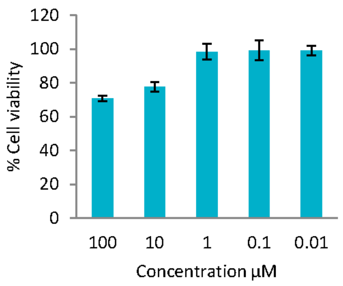

TK-10

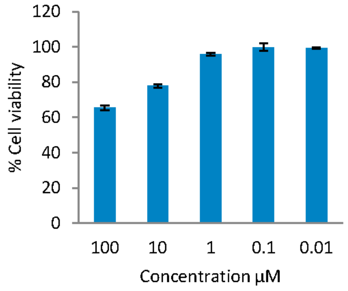

UACC-62

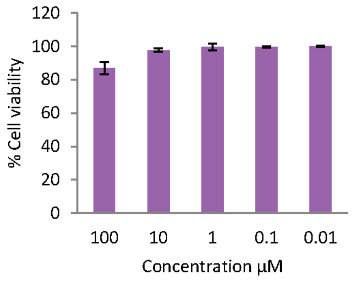

MCF-7

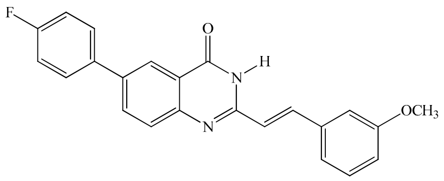

6h

Table S13. Percentage cell viability of TK-10, UACC-62 and MCF-7 cells exposed to different concentrations of **6h**.

Conc. (µM)	Log Conc.	%Viability TK-10	SD	%Viability UACC-62	SD	%Viability MCF-7	SD
100	2	29.71	0.27	35.99	0.05	78.05	0.06
10	1	93.09	3.95	86.12	2.19	99.20	1.34
1	0	99.14	0.44	98.64	0.79	100	1.23
0.1	−1	100	1.71	100	0.23	100	1.19
0.01	−2	100	1.97	100	1.14	100	1.01

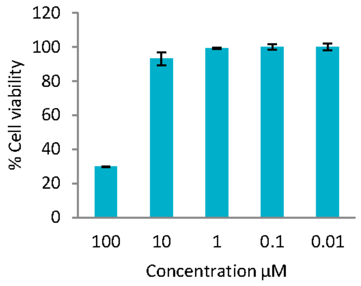

TK-10

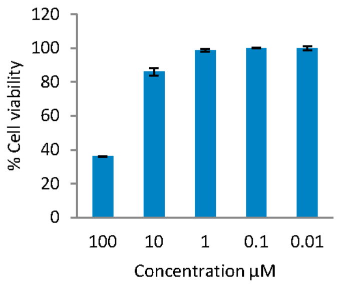

UACC-62

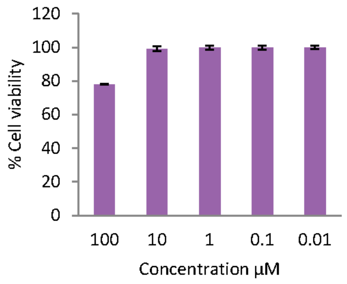

MCF-7
